# Movement Disorders in Scrub Typhus: A Systematic Review

**DOI:** 10.5334/tohm.1148

**Published:** 2026-03-31

**Authors:** Ravindra Kumar Garg, Shweta Pandey, Amita Jain, Ritu Karoli, Vinay Suresh, Sanjay Singhal

**Affiliations:** 1 Department of Neurology, Era’s Lucknow Medical College & Hospital, Era University, Lucknow, India; 2 Department of Neurology, King George’s Medical University, Lucknow, India; 3 All India Institute of Medical Sciences, Rae Bareli, Uttar Pradesh, India; 4 Department of Medicine, Dr RML Institute of Medical Sciences, Lucknow, India; 5 Department of Psychiatry, Warne ford Hospital, University of Oxford, United Kingdom; 6 Department of Pulmonary Medicine, TS Misra Medical College, Lucknow, India

**Keywords:** Ataxia, Opsoclonus–myoclonus, Orientia tsutsugamushi, Rickettsial Diseases

## Abstract

**Background:**

Scrub typhus is a major rickettsial infection in Asia, occasionally presenting with movement disorders due to central nervous system involvement. This systematic review aimed to summarize their frequency, spectrum, diagnostic profile, treatment, and outcomes.

**Methods:**

A comprehensive search of PubMed, Embase, Scopus, and Google Scholar was conducted up to August 2025. All case reports, case series, and cohort studies reporting movement disorders associated with scrub typhus were included. Data were extracted and analyzed descriptively. The protocol was prospectively registered with PROSPERO under the identifier PROSPERO 2025 CRD420251156525.

**Results:**

Across 55 published cases, scrub typhus–associated movement disorders occurred predominantly in young adults (median age 28 years), with a male predominance (61.8%). Opsoclonus and related ocular motor disorders were the most frequent manifestations (41.8%), followed by cerebellar syndromes (34.5%), parkinsonism (12.7%), myoclonus (7.3%), and rare presentations such as ballismus, dystonia, opisthotonus, and akinetic mutism (7.3%). Associated neurological signs included cerebellar involvement (32.7%), altered sensorium (25.5%), extrapyramidal features (21.8%), and seizures (10.9%). In cohort studies including 2437 patients, 63 (2.6%) developed movement disorders, with opsoclonus–myoclonus seen in 22 (34.9%), ataxia in 17, myoclonus in 11 (17.4%), and extrapyramidal features in 25 (39.6%). Rare hyperkinetic manifestations such as hemiballismus and choreoathetoid movements were occasionally reported. Most patients had favorable outcomes, with complete recovery in 38 (69.1%), near-complete recovery in 6 (10.9%), partial recovery in 5 (9.1%), and death in 1 (1.8%).

**Conclusions:**

Movement disorders in scrub typhus, though uncommon, display a wide clinical spectrum and are often reversible.

## Introduction

Scrub typhus is an acute febrile illness caused by *Orientia tsutsugamushi*, an obligate intracellular, Gram-negative bacterium transmitted to humans by the bite of larval trombiculid mites (chiggers) [1]. It is one of the most important rickettsial infections in the Asia-Pacific region, known as the “tsutsugamushi triangle,” which spans from northern Japan and far-eastern Russia to northern Australia and extends westward to Pakistan and Afghanistan. Recent data suggest that more than one billion people live in endemic areas, with an estimated annual incidence of over one million cases, making scrub typhus a significant public health problem in tropical and subtropical regions. Once considered largely confined to rural Asia, the disease is increasingly recognized globally [2,3].

The clinical presentation of scrub typhus is highly variable, ranging from a self-limited febrile illness to severe, life-threatening multi-organ involvement. Classical features include fever, rash, lymphadenopathy, and the presence of a characteristic eschar at the site of the chigger bite. However, in many patients, the disease progresses to involve multiple organ systems, including the respiratory, cardiovascular, renal, and central nervous system (CNS). Neurological involvement occurs in approximately 10–20% of hospitalized cases and may manifest as meningitis, meningoencephalitis, cranial neuropathies, acute disseminated encephalomyelitis, Guillain–Barré syndrome, or immune-mediated neuropathies. Among these, movement disorders represent a rare but clinically significant subset of neurological manifestations that remain underrecognized and poorly characterized [2,4,5,6].

Movement disorders associated with scrub typhus encompass a heterogeneous spectrum, including parkinsonism, chorea, dystonia, myoclonus, opsoclonus–myoclonus syndrome, tremor, hemiballismus, and cerebellar ataxia. They may present acutely during the systemic phase of the infection or emerge as post-infectious, para- or post-immune phenomena weeks after the initial illness. Histopathological evidence from experimental models and human autopsies supports the role of widespread microglial activation, perivascular inflammation, and small-vessel vasculitis in the neuropathogenesis of the disease. The emergence of movement disorders in this context may reflect localized involvement of extrapyramidal structures, particularly the basal ganglia, thalamus, cerebellum, or brainstem [7,8,9].

Despite their clinical importance, these manifestations are often overlooked or misdiagnosed due to their rarity, diverse presentation, and the lack of clinician awareness. Most of the available evidence is scattered across isolated case reports or small case series, limiting our understanding of the true incidence, temporal profile, underlying mechanisms, diagnostic features, treatment approaches, and prognostic outcomes. Furthermore, the clinical spectrum may be broader than currently recognized, and subtle or transient movement disorders may go undetected without systematic evaluation. Timely recognition is crucial, as many of these manifestations are potentially reversible with appropriate antimicrobial therapy and, in immune-mediated cases, may respond to immunomodulatory treatment.

The objectives, of this review, are to delineate the full clinical spectrum of these neurological manifestations, elucidate the underlying pathophysiological mechanisms proposed in the literature, evaluate the therapeutic strategies employed, and summarize reported patient outcomes.

## Methods

This systematic review was conducted following the Preferred Reporting Items for Systematic Reviews and Meta-Analyses (PRISMA) recommendations. A predefined protocol was developed to guide the search strategy, study selection, data extraction, and synthesis processes. The protocol was prospectively registered with PROSPERO under the identifier PROSPERO 2025 CRD420251156525 [10].

### Search Strategy

A comprehensive literature search was conducted in PubMed, Embase, Scopus, and Google Scholar from database inception to September 27, 2025. The full database-specific search strings are provided in Table 1, and screening of the first 50 pages of Google Scholar results was performed in line with commonly adopted systematic review practices to capture relevant grey literature while maintaining feasibility, as results beyond this range are typically less relevant. Reference lists of all included studies and relevant review articles were also manually screened to identify further eligible reports. No language restrictions were applied, but only studies providing sufficient clinical detail to enable extraction of relevant data were included.

**Table 1:** Search Strategy.


**PubMed**	(“Scrub Typhus”[Mesh] OR “Orientia tsutsugamushi”[tiab] OR “tsutsugamushi disease”[tiab] OR “scrub typhus”[tiab] OR “Orientia infection”[tiab] OR (rickettsial[tiab] AND “scrub typhus”[tiab])) AND (“Movement Disorders”[Mesh] OR “Neurologic Manifestations”[Mesh] OR “movement disorder”[tiab] OR neurological[tiab] OR neurology[tiab] OR “gait disorder”[tiab] OR tremor[tiab] OR myoclonus[tiab] OR ataxia[tiab] OR parkinsonism[tiab] OR dystonia[tiab] OR chorea[tiab] OR ballism[tiab] OR hemiballismus[tiab] OR hemiballism[tiab] OR opsoclonus[tiab] OR hyperkinesia[tiab] OR hypokinesia[tiab] OR extrapyramidal[tiab] OR “tic disorder”[tiab] OR tics[tiab] OR stereotypy[tiab] OR stereotypies[tiab] OR automatisms[tiab] OR akathisia[tiab] OR blepharospasm[tiab] OR rigidity[tiab] OR dyskinesia[tiab])

**Embase**	(“scrub typhus”/exp OR “orientia tsutsugamushi”:ti,ab OR “tsutsugamushi disease”:ti,ab OR “scrub typhus”:ti,ab OR “orientia infection”:ti,ab OR (rickettsial:ti,ab AND “scrub typhus”:ti,ab)) AND (“movement disorder”/exp OR “neurologic manifestation”/exp OR “movement disorder”:ti,ab OR neurological:ti,ab OR neurology:ti,ab OR “gait disorder”:ti,ab OR tremor:ti,ab OR myoclonus:ti,ab OR ataxia:ti,ab OR parkinsonism:ti,ab OR dystonia:ti,ab OR chorea:ti,ab OR ballism:ti,ab OR hemiballismus:ti,ab OR hemiballism:ti,ab OR opsoclonus:ti,ab OR hyperkinesia:ti,ab OR hypokinesia:ti,ab OR extrapyramidal:ti,ab OR “tic disorder”:ti,ab OR tics:ti,ab OR stereotypy:ti,ab OR stereotypies:ti,ab OR automatisms:ti,ab OR akathisia:ti,ab OR blepharospasm:ti,ab OR rigidity:ti,ab OR dyskinesia:ti,ab)

**Scopus**	(TITLE-ABS-KEY(“scrub typhus” OR “Orientia tsutsugamushi” OR “tsutsugamushi disease” OR “Orientia infection” OR (rickettsial AND “scrub typhus”))) AND (TITLE-ABS-KEY(“movement disorder” OR “movement disorders” OR neurological OR neurology OR “gait disorder” OR tremor OR myoclonus OR ataxia OR parkinsonism OR dystonia OR chorea OR ballism OR hemiballismus OR hemiballism OR opsoclonus OR hyperkinesia OR hypokinesia OR extrapyramidal OR “tic disorder” OR tics OR stereotypy OR stereotypies OR automatisms OR akathisia OR blepharospasm OR rigidity OR dyskinesia))

**Google Scholar**	(“scrub typhus” OR “Orientia tsutsugamushi” OR “tsutsugamushi disease”) AND (“movement disorder” OR tremor OR myoclonus OR ataxia OR parkinsonism OR dystonia OR chorea OR opsoclonus OR dyskinesia OR hyperkinesia OR extrapyramidal)


### Eligibility Criteria

We included published case reports, case series, and observational studies describing patients of any age with confirmed or probable scrub typhus infection and a clinically diagnosed movement disorder occurring in temporal association with the illness. Confirmation of scrub typhus was accepted if based on one or more of the following: a positive serological test such as immunoglobulin M enzyme-linked immunosorbent assay, indirect immunofluorescence assay, or Weil–Felix test; molecular detection by polymerase chain reaction (PCR); presence of a characteristic eschar and compatible clinical syndrome; or a consistent clinical picture supported by epidemiological exposure. The presence of an eschar, when identified, was regarded as a highly specific diagnostic clue that significantly strengthens the clinical diagnosis of scrub typhus, especially in endemic areas and in patients presenting with nonspecific systemic manifestations [11,12,13].

Studies were excluded if they did not provide individual patient-level data, lacked sufficient diagnostic confirmation, were animal or in vitro studies, were narrative reviews or editorials, or were conference abstracts without adequate clinical information.

### Definitions

Movement disorders were classified according to the accepted clinical definitions of the International Parkinson and Movement Disorder Society. These included parkinsonism, chorea, dystonia, tremor, myoclonus, opsoclonus–myoclonus syndrome, ataxia, tics, stereotypies, hemiballismus, and other hyperkinetic or hypokinetic extrapyramidal syndromes. Cerebellar involvement manifesting as ataxia or dysmetria was also included. Movement disorders were categorized according to the predominant phenomenological syndrome. Associated neurological findings represent additional neurological examination signs accompanying the movement disorder, such as cerebellar signs (like nystagmus, dysmetria, hypotonia), extrapyramidal signs (rigidity or bradykinesia), cranial nerve palsies, pyramidal signs, or seizures. Because multiple signs may coexist in a single patient, these categories were not mutually exclusive. For consistency, historical terms reported in older literature were cross-referenced and reclassified according to current diagnostic nomenclature [14].

### Study Selection

All references retrieved from the database searches were imported into EndNote 21 (Clarivate Analytics, Philadelphia, United States) for duplicate removal. Two reviewers (AJ and VS) independently screened the titles and abstracts of all retrieved citations. Full-text articles were then obtained for all studies that met the inclusion criteria or where eligibility could not be determined from the abstract alone. Full texts were assessed independently by two reviewers (RKG and SP) for final inclusion. Any disagreements were resolved through consensus. The study selection process adhered to PRISMA guidelines and was documented in a PRISMA flow diagram.

### Data Extraction

A standardized and piloted data extraction form was used to collect information from each eligible study. Data were independently extracted by two reviewers and included the following variables: first author, year of publication, country, patient age and sex, immune status, systemic features, presence of eschar, duration of illness, diagnostic method, onset of neurological manifestations relative to infection (acute or post-infectious), type of movement disorder, associated neurological signs, cerebrospinal fluid findings, neuroimaging results, electrophysiological data (where available), treatment administered (antibiotic, immunotherapy, and symptomatic therapy), clinical response, outcomes, follow-up duration, and proposed pathophysiological mechanisms.

### Quality Assessment

The methodological quality of included case reports and case series was assessed using the tool proposed by Murad and colleagues, which evaluates four domains: selection, ascertainment, causality, and reporting [15]. Each study was rated as “good,” “fair,” or “poor” based on the number of domains adequately addressed [16]. Assessments were performed independently by two reviewers, with disagreements resolved through consensus. The quality of cohort or observational studies were not assessed for quality.

### Data Synthesis

Extracted data were summarized descriptively. Categorical variables were presented as frequencies and percentages, whereas continuous variables were expressed as mean, median, range, and interquartile range, depending on the availability of data. Reported cases were categorized based on the type of movement disorder into hyperkinetic, hypokinetic, and cerebellar syndromes. Patterns of neuroimaging findings, associated systemic and neurological manifestations, treatment modalities, and clinical outcomes were analyzed within these subgroups. The results were synthesized narratively, and trends in clinical presentation, timing, proposed mechanisms, and prognosis were identified and discussed.

## Results

**Figure 1:**
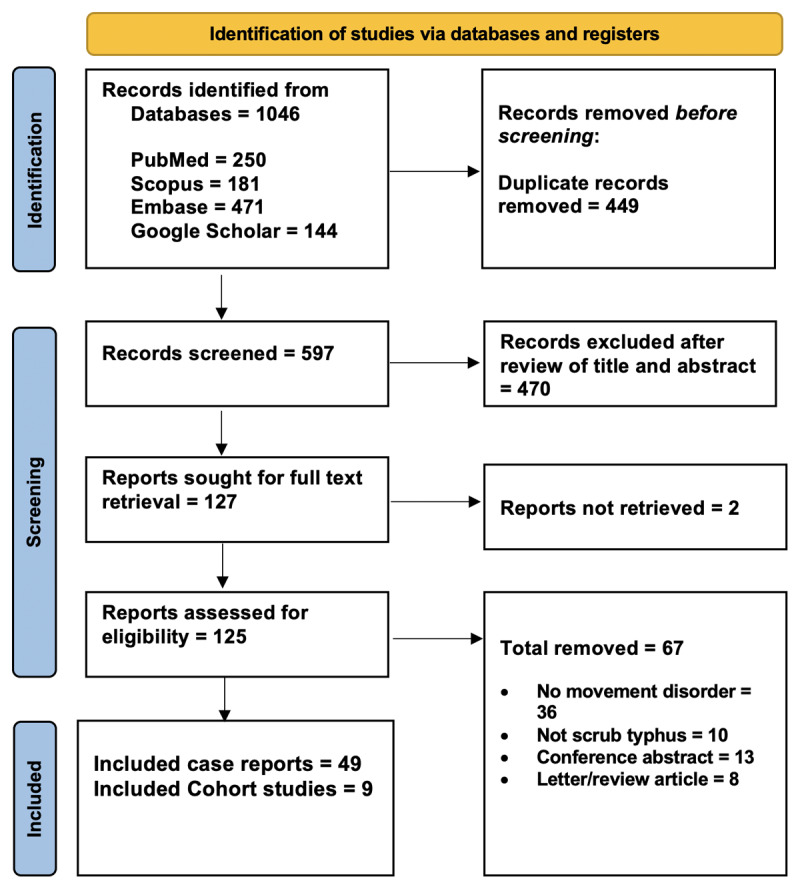
PRISMA 2020 flow diagram summarizing study selection: 1046 records identified, 449 duplicates removed, 597 screened, 125 assessed, and 58 studies included, comprising 49 case reports and 9 cohort studies.

A total of 55 published cases of scrub typhus–associated movement disorders were analyzed. All 55 cases were rated as good quality (Supplementary Item 1). The PRISMA checklist is provided as Supplementary Item 2. The PRISMA flow chart depicts article selection process (Figure 1). We also analysed 9 cohort studies. Details of the extracted data are provided in Supplementary Tables.

The median age of patients with scrub typhus–associated movement disorders was 28 years (range: 3–73), with males constituting 61.8% of cases. Most reports originated from India (85.7%), followed by South Korea and a few other Asian countries. Fever was present in all patients, and neurological manifestations most commonly appeared 6–10 days after illness onset (23, 41.8%), and overall 41 patients (74.5%) developed neurological symptoms within the first 10 days of illness. Diagnosis was confirmed primarily by IgM ELISA (55.6%), while advanced modalities such as PCR (5.6%) and next-generation sequencing (1.9%) were infrequently used. A characteristic eschar was present in 38.2% of patients, while 30.9% lacked this finding. Other systemic manifestations included headache and vomiting (36.4% each), liver involvement such as transaminitis or hepatomegaly (30.9%), myalgia or arthralgia (18.2%), respiratory involvement or acute respiratory distress syndrome (10.9%), renal involvement (10.9%), thrombocytopenia and coagulopathy (10.9%), and hypotension or multiorgan dysfunction (7.3%). Less common features included rash (7.3%), papilledema or photophobia (7.3%), and lymphadenopathy (5.5%). Neuroimaging was normal in 61.8% of cases but revealed cerebellar hyperintensities, basal ganglia or thalamic involvement, or meningeal enhancement in others. CSF findings typically showed lymphocytic pleocytosis or elevated protein (Table 2).

**Table 2:** Baseline characteristics and clinical features of patients with scrub typhus–associated movement disorders (n = 55).


VARIABLES	VALUE

**Age (in years)**	NA: 3 Mean: 31.8 Median: 28 Range: 3–73 IQR: 28.5

**Sex**	Male: 34 (61.8%) Female: 20 (36.4%) NA: 1 (1.8%)

**Country-wise distribution of published articles (n = 49)**	India: 42 (85.7%) South Korea: 3 (6.1%) China: 1 (2.0%) Nepal: 1 (2.0%) Sri Lanka: 1 (2.0%) Taiwan: 1 (2.0%)

**Diagnostic Serology/CSF**	NA: 1(1.9%) IgM ELISA: 30 (55.6%) IgM ELISA + Weil–Felix/combo: 6 (11.1%) Weil–Felix test: 4 (7.4%) Indirect immunofluorescence assay (IFA): 4 (7.4%) PCR (molecular test): 3 (5.6%) Rapid antibody test: 2 (3.7%) Next-generation sequencing (NGS): 1 (1.9%) Other serology (unspecified): 4 (7.4%)

**Duration of illness**	≤5 days: 18 (32.7%) 6–10 days: 23 (41.8%) 11–15 days: 6 (10.9%) >15 days: 4 (7.3%) Not reported: 4 (7.3%)

**Type of Movement Disorder/Ataxia**	Opsoclonus and related ocular motor disorders: 23 (41.8%) Cerebellar ataxia and cerebellar syndromes: 19 (34.5%) Parkinsonism: 7 (12.7%) Myoclonus and related hyperkinetic disorders: 4 (7.3%) Other movement disorders (ballismus, dystonia, opisthotonus, akinetic mutism): 4 (7.3%)

Associated Neurological Findings*	Other cerebellar signs (nystagmus, dysmetria, hypotonia; excluding ataxia): 18 (32.7%) Extrapyramidal signs (bradykinesia, rigidity, tremor): 12 (21.8%) Seizures/status epilepticus: 6 (10.9%) Cranial nerve palsies/brainstem involvement: 8 (14.5%) Meningeal signs/raised intracranial pressure: 9 (16.4%) Pyramidal signs (spasticity, quadriparesis, hyperreflexia): 7 (12.7%) Peripheral neuropathy/sensory deficits: 3 (5.5%)

**Other Clinical Features**	Fever: 55 (100%) Headache: 20 (36.4%) Vomiting/GI symptoms: 20 (36.4%) Myalgia/Arthralgia/Malaise: 10 (18.2%) Altered sensorium, confusion, drowsiness, akinetic mutism: 14 (25.5%) Liver involvement (transaminitis/hepatomegaly/jaundice): 17 (30.9%) Thrombocytopenia/Coagulopathy/Bleeding: 6 (10.9%) Rash/Petechiae/Erythema: 4 (7.3%) Respiratory involvement/ARDS: 6 (10.9%) Renal involvement/AKI: 6 (10.9%) Papilledema/Photophobia/Meningeal signs: 4 (7.3%) Lymphadenopathy/Episcleritis: 3 (5.5%) Hypotension/Shock/MODS: 4 (7.3%) Other neurological signs (rigidity, tremor, imbalance): 5 (9.1%)

**Presence of a characteristic eschar**	Present: 21 (38.2%) Absent: 17 (30.9%) Not reported/Not mentioned/Not specified: 17 (30.9%)


*Movement disorder categories represent the primary motor syndrome, whereas associated neurological findings denote additional examination signs; therefore, categories are not mutually exclusive.

Movement disorders showed a wide clinical spectrum. Opsoclonus and related ocular motor disturbances were most frequent (41.8%), followed by cerebellar syndromes (34.5%), parkinsonism (12.7%), myoclonus (7.3%), and rarer forms such as ballismus, dystonia, opisthotonus, and akinetic mutism (7.3%). Associated neurological findings included cerebellar signs (32.7%), altered sensorium (25.5%), extrapyramidal signs (21.8%), meningeal signs (16.4%), cranial nerve palsies (14.5%), seizures (10.9%), pyramidal signs (12.7%), and peripheral neuropathy (5.5%) (Table 2).

Among 55 reported cases, isolated cerebellar ataxia was the most frequent presentation, observed in 17 patients (30.9%), followed by isolated opsoclonus or ocular motor disorders in 9 (16.4%), isolated parkinsonism in 4 (7.3%), and isolated myoclonus in 3 (5.5%). Combined movement disorders occurred in 11 patients (20.0%), most commonly opsoclonus–myoclonus with ataxia or tremor, and rarely parkinsonism with myoclonus or choreiform movements. Parkinsonism was typically akinetic–rigid, with bilateral bradykinesia, rigidity, masked facies, and resting tremor. Myoclonus was usually multifocal or generalized, including one case of cortical multifocal myoclonus and another of diaphragmatic myoclonus. In most patients, movement disorders developed acutely during the febrile phase, usually within 5–10 days of illness onset, whereas a smaller proportion occurred post-infectiously during early recovery (Table 2).

Most patients had favorable outcomes, with complete recovery in 38 (69.1%) cases. Near-complete recovery with minimal residual deficits occurred in 6 (10.9%), while partial recovery with persistent neurological deficits was reported in 5 (9.1%) patients. One patient (1.8%) died, and outcome details were not available in four cases. Follow-up duration ranged from 2 weeks to more than 1 year, with longer follow-up reported in patients with persistent deficits. Treatment primarily consisted of antibiotics alone or combined with corticosteroids or immunotherapy, while symptomatic therapies such as antiepileptics or benzodiazepines were occasionally used for myoclonus or tremor with partial benefit. Overall, complete resolution of movement disorders occurred in 37 (67.3%), partial improvement in 9 (16.4%), and death in 1 (1.8%), supporting the largely reversible and para-infectious nature of these neurological manifestations when appropriately treated (Table 3).

**Table 3:** Clinical investigations, treatment, and outcomes in scrub typhus–associated movement disorders (n = 55).


VARIABLES	VALUES

**Blood investigations**	Thrombocytopenia: 27 (49.1%) Elevated liver enzymes/transaminitis/hyperbilirubinemia: 23 (41.8%) Leukocytosis/leukopenia/lymphocytosis: 16 (29.1%) Hyponatremia/electrolyte imbalance: 6 (10.9%) Renal dysfunction/elevated urea-creatinine: 6 (10.9%) Elevated inflammatory markers (CRP, ESR, ferritin): 7 (12.7%) Anemia/hypoalbuminemia: 5 (9.1%) Normal findings: 3 (5.5%) Not reported: 7 (12.7%)

**CSF findings**	Lymphocytic pleocytosis ± elevated protein: 26 (47.3%) Elevated protein with normal or few cells (albumin-cytologic dissociation): 5 (9.1%) Normal CSF findings: 10 (18.2%) Low glucose (with pleocytosis/protein elevation): 4 (7.3%) Acellular CSF: 2 (3.6%) Not performed/Not reported: 8 (14.5%)

**Neuroimaging Findings**	Normal imaging: 34 (61.8%) Cerebellar involvement (hyperintensity, edema, enhancement): 7 (12.7%) Basal ganglia/thalamic/brainstem lesions: 4 (7.3%) Leptomeningeal enhancement/pachymeningeal enhancement: 3 (5.5%) Intracranial hemorrhage/SAH/IVH: 1 (1.8%) Gliosis/demyelination/chronic changes: 3 (5.5%) Other findings (calcified granuloma, hydrocephalus, ARDS findings only): 3 (5.5%)

**Treatment**	Antibiotics only (mainly doxycycline ± azithromycin/ceftriaxone): 27 (49.1%) Antibiotics + corticosteroids (dexamethasone/methylprednisolone): 10 (18.2%) Antibiotics + immunotherapy (IVIG and/or steroids): 5 (9.1%) Antibiotics + symptomatic/supportive therapy (antiepileptics, ICP control, etc.): 8 (14.5%) Supportive/symptomatic therapy only: 2 (3.6%) Not reported: 3 (5.5%)

**Response on movement disorder**	Complete resolution of movement disorders: 37 (67.3%) Partial improvement with residual signs (e.g., mild tremor, persistent ataxia, parkinsonism): 9 (16.4%) Gradual or near-complete improvement over weeks to months: 4 (7.3%) Spontaneous resolution without specific therapy: 2 (3.6%) No improvement/fatal outcome: 1 (1.8%) Not reported: 2 (3.6%)

**Outcome**	Complete recovery/full resolution: 38 (69.1%) Near-complete recovery with minimal residual signs: 6 (10.9%) Partial recovery with persistent deficits: 5 (9.1%) Spontaneous resolution: 1 (1.8%) Death: 1 (1.8%) Not reported/insufficient details: 4 (7.3%)

**Duration of follow up**	≤2 weeks: 10 (18.2%) 2–4 weeks: 8 (14.5%) 1–3 months: 10 (18.2%) 4–6 months: 2 (3.6%) ≥1 year: 5 (9.1%) Not reported/not specified: 20 (36.4%)


ARDS – Acute Respiratory Distress Syndrome; CRP – C-Reactive Protein; CSF – Cerebrospinal Fluid; ESR – Erythrocyte Sedimentation Rate; ICP – Intracranial Pressure; IVH – Intraventricular Hemorrhage; IVIG – Intravenous Immunoglobulin; SAH – Subarachnoid Hemorrhage.

Immune-mediated or para-infectious mechanisms were the most frequently proposed (21 (38.2%)), followed by endothelial invasion and vasculitic injury (10 (18.2%)). Other mechanisms included disruption of specific neuronal circuits (8 (14.5%)), combined immune and vascular injury (6 (10.9%)), autoimmune demyelination (4 (7.3%)), direct CNS invasion (3 (5.5%)), and cytokine-mediated dopaminergic dysfunction (3 (5.5%)). These mechanisms are discussed alongside the clinical phenotypes and imaging findings, and their categorization is summarized in the Table 4.

**Table 4:** Proposed Mechanisms of Movement Disorders in Scrub Typhus (n = 55).


MECHANISM	DESCRIPTION	CASES (N)	PERCENTAGE (%)

Immune-mediated/para-infectious response	Antibody-mediated cross-reactivity, cytokine release, and neuroinflammation triggered by O. tsutsugamushi, leading to opsoclonus–myoclonus, cerebellitis, basal ganglia dysfunction, or post-infectious encephalitis.	21	38.2%

Endothelial invasion and vasculitis	Direct invasion of vascular endothelium causes vasculitis, perivasculitis, cytokine storm, and microglial activation, leading to parenchymal injury, cerebellar involvement, or intracerebral hemorrhage.	10	18.2%

Combined immune and vascular injury	Interaction of antibody-mediated injury with vasculitic endothelial damage, often producing mixed features like opsoclonus with vasculitic lesions or cerebellitis with microvascular injury.	6	10.9%

Direct CNS invasion and neuronal damage	Direct penetration of the pathogen into brain tissue (thalamus, brainstem, basal ganglia) causing structural neuronal damage and subsequent movement disorders.	3	5.5%

Autoimmune demyelination/ADEM-like response	Molecular mimicry and cytokine-mediated inflammation lead to CNS demyelination, Guillain–Mollaret triangle disruption, or hypertrophic olivary degeneration.	4	7.3%

Disruption of specific neuronal circuits	Dysfunction of omnipause neurons, Purkinje cells, or saccadic burst neurons in the pontine reticular formation and cerebellar fastigial nucleus results in opsoclonus, ocular flutter, or abnormal motor control.	8	14.5%

Cytokine-mediated dopaminergic disruption	Pro-inflammatory cytokines (e.g., IL-8, MCP-1, MIP-1α/β) activate microglia and damage dopaminergic neurons, contributing to parkinsonism and related movement disorders.	3	5.5%


ADEM – Acute Disseminated Encephalomyelitis; CNS – Central Nervous System; IL-8 – Interleukin-8; MCP-1 – Monocyte Chemoattractant Protein-1; MIP-1α – Macrophage Inflammatory Protein-1 alpha; MIP-1β – Macrophage Inflammatory Protein-1 beta; OMS – Opsoclonus–Myoclonus Syndrome.

### Cohort studies

Across nine studies including 2437 patients with scrub typhus, 63 patients (2.6%) developed movement disorders. The reported frequency varied from 1.09% in large cohorts to 100% in a small pediatric series. Ataxia and cerebellar syndromes were among the most frequent manifestations, reported in 4 of 9 studies and affecting at least 17 patients. Opsoclonus and opsoclonus-myoclonus occurred in approximately 22 patients (34.9%), while myoclonus alone was seen in at least 11 cases (17.4%). Extrapyramidal manifestations such as tremor, rigidity, and bradykinesia were documented in about 25 patients (39.6%), with tremor reported in 8 cases, rigidity in 11, and parkinsonian gait in 6. Rare hyperkinetic features were observed in smaller numbers, including hemiballismus (1), choreoathetoid movements (1), perioral dyskinesia (1), lip smacking (1), teeth grinding (1), and rapid eye blinking (1). Overall, the prevalence of movement disorders ranged between 1.2% and 18% in most cohorts, highlighting a heterogeneous but clinically significant neurological complication (Supplementary Table 2).

## Discussion

Scrub typhus–associated movement disorders present a broad and heterogeneous clinical spectrum, with opsoclonus–myoclonus being one of the most characteristic and frequently reported manifestations, often highlighting underlying neurological involvement. These abnormalities frequently occur alongside other features such as encephalopathy, cranial neuropathies, seizures, pyramidal signs, and meningeal irritation, reflecting widespread CNS involvement. The spectrum includes both hypo- and hyperkinetic disorders, frequently coexisting with systemic complications such as hepatic dysfunction, hematological abnormalities, and renal impairment, underscoring the multisystem nature of the disease. In many patients, movement abnormalities, including opsoclonus–myoclonus, emerge early or appear as the sole neurological presentation, serving as an important diagnostic clue in endemic settings. Neuroimaging is often normal, although selective changes involving deep grey matter structures or the cerebellum may be detected, while cerebrospinal fluid findings commonly reveal inflammatory changes. The temporal profile is variable, ranging from acute to delayed presentations, and clinical outcomes are generally favourable with early antimicrobial and supportive therapy. The diversity of movement disorder phenotypes, particularly the frequent occurrence of opsoclonus–myoclonus and their association with multisystem involvement, emphasizes their diagnostic significance and clinical relevance in scrub typhus.

Movement disorders in scrub typhus originate from structural and functional disruptions across the motor network. Basal ganglia involvement- especially of the caudate, putamen, and globus pallidus-has been associated in case reports with parkinsonism, rigidity, tremor, and other extrapyramidal signs. The cerebellum and its nuclei (e.g. dentate, fastigial) are also implicated, manifesting as coordination deficits, gait instability, and cerebellar ataxia [9,17,18]. Brainstem structures, including the pontine reticular formation, omnipause neurons, and saccadic burst circuits, are critical for ocular motor control and help explain syndromes such as opsoclonus–myoclonus in scrub typhus [18,19]. Lesions in the thalamus or subthalamic nucleus may underlie hyperkinetic disorders such as chorea or hemiballismus [9]. Injury to corticospinal tracts and cerebello-thalamo-cortical circuits further broadens the motor phenotype.

**Figure 2:**
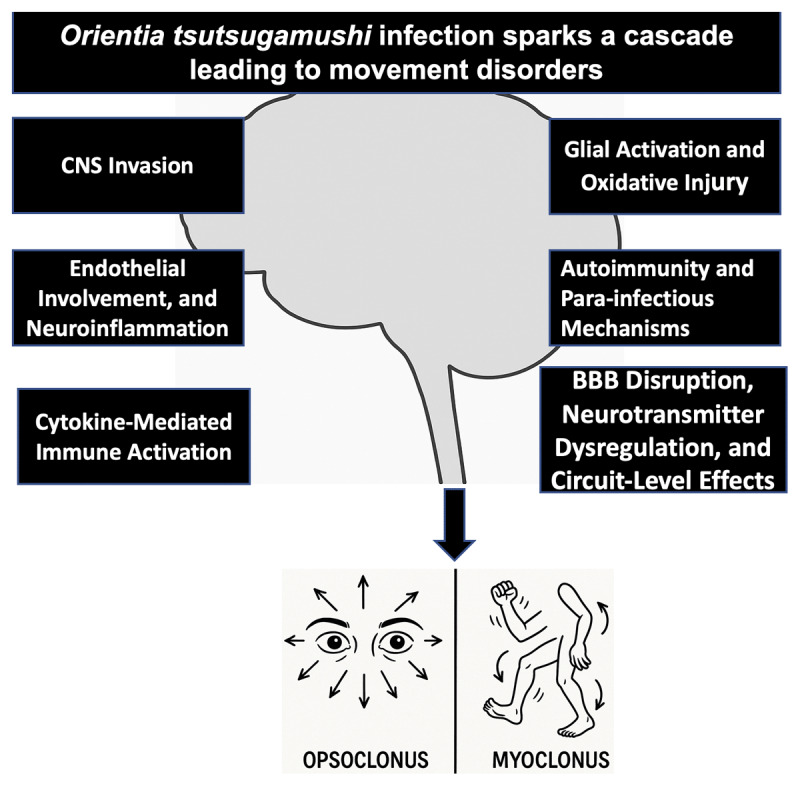
Mechanisms linking *Orientia tsutsugamushi* infection to movement disorders, including CNS invasion, neuroinflammation, immune activation, and blood–brain barrier disruption, leading to opsoclonus and myoclonus.

The mechanisms underlying movement disorders in scrub typhus likely reflect a complex interplay of infectious, vascular, immune, and neuroinflammatory processes affecting key motor control circuits within the CNS (Table 2). Early neuropathological observations demonstrated that *Orientia tsutsugamushi* infection can extend beyond systemic involvement to affect the CNS. Autopsy findings revealed perivascular mononuclear infiltration, vasculitis, and focal parenchymal lesions, suggesting that the pathogen is capable of breaching the blood–brain barrier (BBB) and contributing to encephalitic injury. Although direct neuronal infection was not conclusively demonstrated, these findings indicate that scrub typhus may enter the CNS and initiate inflammatory injury within neural tissue [20] (Figure 2).

Experimental studies further suggest that once *Orientia tsutsugamushi* reaches the CNS, it preferentially localizes to endothelial and immune cell populations rather than neurons. This cellular tropism leads to endothelial activation, BBB disruption, and recruitment of inflammatory mediators and immune cells, producing a robust neuroinflammatory response [21,22]. Endothelial dysfunction and small-vessel vasculitis may exacerbate perivascular inflammation, resulting in microinfarctions and focal ischemia. Such vascular injury can disrupt motor pathways involving the basal ganglia, cerebellum, and brainstem, thereby contributing to clinical manifestations such as parkinsonism, chorea, and dystonia [20,23].

Cytokine-mediated immune activation also appears to play an important role. Infection with *Orientia tsutsugamushi* induces the release of pro-inflammatory cytokines, including interleukin-6, tumor necrosis factor-alpha, and interferon-gamma, which recruit additional immune cells to the CNS, alter neuronal signaling, and disturb neurotransmitter homeostasis [21,24,25,26]. Activated microglia and astrocytes further amplify this inflammatory cascade through the production of reactive oxygen species and nitric oxide, leading to oxidative stress and neuronal injury that can impair motor circuits and contribute to the heterogeneity of movement disorders observed in this condition [21,24,27,28].

In addition, immune-mediated para-infectious mechanisms may be important in some patients. Molecular mimicry between *Orientia tsutsugamushi* antigens and host neuronal proteins may trigger the production of cross-reactive antibodies, leading to autoimmune neurological syndromes such as autoimmune encephalitis, opsoclonus–myoclonus, or basal ganglia dysfunction [29]. BBB disruption facilitates the entry of immune cells and inflammatory mediators into the CNS, intensifying local neuroinflammatory cascades and producing secondary neuronal injury. Combined with small-vessel vasculitis and perivascular inflammation, these processes may result in focal ischemic or hemorrhagic lesions affecting basal ganglia, cerebellar, or brainstem circuits [21,23]. The resulting neuronal injury and inflammatory modulation of synaptic transmission may disrupt dopaminergic and GABAergic signaling, further impairing motor control networks and giving rise to the diverse spectrum of movement disorders observed in scrub typhus [6,25].

This systematic review has several limitations. Most included studies were case reports, small case series, or retrospective cohorts, which are inherently prone to reporting bias, incomplete data, and heterogeneity in clinical descriptions. Diagnostic heterogeneity was substantial across studies, with considerable variability in the criteria and methods used to diagnose scrub typhus and associated neurological manifestations. Differences in serological assays, molecular tests, and neuroimaging approaches, together with small sample sizes, limit the generalizability of the findings and may have influenced case detection. In addition, there was marked geographic clustering of reported cases, with approximately 85% originating from India. This regional predominance may reflect differences in disease recognition, reporting practices, or research activity rather than the true global distribution of scrub typhus–associated movement disorders, thereby limiting broader epidemiological inference. Another important limitation is the lack of standardized neurological examination and reporting across studies. Clinical assessments were often inconsistently described, and detailed neurological phenotyping was frequently lacking, which may have affected accurate characterization of the movement disorder spectrum. Long-term follow-up was infrequently reported, restricting a clear understanding of long-term neurological outcomes. Furthermore, immunological, molecular, and neuropathological investigations were inconsistently performed, leaving the proposed pathogenic mechanisms largely speculative. Publication bias is also likely, as unusual or severe neurological manifestations are more likely to be reported than typical or mild presentations, which may distort the true frequency and clinical spectrum of movement disorders in scrub typhus. Heterogeneity in the reporting of treatments and outcomes further limits firm conclusions regarding prognosis and therapeutic response. Larger prospective studies with standardized diagnostic criteria, uniform neurological examination protocols, and systematic follow-up across diverse geographic regions are needed to better define the clinical spectrum, mechanisms, and outcomes of these disorders.

In conclusion, scrub typhus–associated movement disorders, particularly opsoclonus–myoclonus, represent important but under-recognized neurological manifestations with diverse clinical presentations. They often occur alongside systemic and neurological complications and may serve as key diagnostic clues. Early recognition and prompt treatment are crucial for favourable outcomes and minimizing long-term neurological sequelae.

## Use of Artificial Intelligence (AI) Software

ChatGPT 5 was used to correct English and Grammar. During the preparation of this work the authors used ChatGPT 5 in order to correct English and Grammar along with data analysis. After using this tool/service, the authors reviewed and edited the content as needed and take full responsibility for the content of the published article.

## Data Accessibility Statement

All data generated or analyzed during this study are provided in the published article and its supplementary materials.

## Additional Files

The additional files for this article can be found as follows:

10.5334/tohm.1148.s1 Supplementary Item 1. Quality Assessment.

10.5334/tohm.1148.s2 Supplementary Item 2. PRISMA checklist.

10.5334/tohm.1148.s3 Supplementary Table 1. Tables 1a–1c.

10.5334/tohm.1148.s4 Supplementary Table 2. Summary of Cohort Studies Reporting Movement Disorders in Scrub Typhus.
