# Supplementary material for: Movement Disorders in Scrub Typhus: A Systematic Review
Source: Tremor Other Hyperkinet Mov (N Y). 2026 Mar 31;16:22. doi: 10.5334/tohm.1148 (PMC13045785; doi:10.5334/tohm.1148)
Supplement: Supplementary Item 1. — Quality Assessment. [file tohm-16-1-1148-s1.pdf]

Supplementary item-1: Movement Disorders in Scrub Typhus: A Systematic Review

QUALITY ASSESSMENT

Reference	Does the patient represent the whole experience of the investigator	Was the exposure adequately ascertained?	Was the outcome adequately ascertained?	Were other alternative causes that may explain the observation ruled out?	Was there a challenge and/or re-challenge phenomenon?	Was there a dose-response effect?	Was follow-up long enough for outcomes to occur?	Is the case(s) described with sufficient details to allow practitioners make inferences related to their own practice?	Score
Salini et al., 2025	**Yes**	**Yes**	**Yes**	**Yes**	**NA**	**Yes**	**Yes**	**No**	**7**
	**Yes**	**Yes**	**Yes**	**Yes**	**NA**	**Yes**	**Yes**	**Yes**	**7**
Saibaba et al., 2025	**Yes**	**Yes**	**Yes**	**Yes**	**NA**	**Yes**	**Yes**	**Yes**	**7**
Biswas et al., 2025	**Yes**	**Yes**	**Yes**	**Yes**	**NA**	**Yes**	**Yes**	**Yes**	**7**
Bahadur et al., 2025	**Yes**	**Yes**	**Yes**	**Yes**	**NA**	**Yes**	**Yes**	**Yes**	**7**
Alam et al., 2025	**Yes**	**Yes**	**Yes**	**Yes**	**NA**	**Yes**	**Yes**	**Yes**	**7**
Thomas et al., 2024	**Yes**	**Yes**	**Yes**	**Yes**	**NA**	**Yes**	**Yes**	**Yes**	**7**
Singh & Singh, 2024	**Yes**	**Yes**	**Yes**	**Yes**	**NA**	**No**	**No**	**Yes**	**7**
Reddy et al., 2024	**Yes**	**Yes**	**Yes**	**Yes**	**NA**	**Yes**	**Yes**	**Yes**	**7**
Puppala et al., 2024	**Yes**	**Yes**	**Yes**	**Yes**	**NA**	**Yes**	**Yes**	**Yes**	**7**

	**Yes**	**Yes**	**Yes**	**Yes**	**NA**	**Yes**	**Yes**	**Yes**	**7**
Neela et al., 2024	**Yes**	**Yes**	**Yes**	**Yes**	**NA**	**Yes**	**Yes**	**Yes**	**7**
Meena et al., 2024	**Yes**	**Yes**	**Yes**	**Yes**	**NA**	**Yes**	**Yes**	**Yes**	**7**
Li et al., 2024	**Yes**	**Yes**	**Yes**	**Yes**	**NA**	**Yes**	**Yes**	**Yes**	**7**
Jafri et al., 2024	**Yes**	**Yes**	**Yes**	**Yes**	**NA**	**Yes**	**Yes**	**Yes**	**7**
Datta et al., 2024	**Yes**	**Yes**	**Yes**	**Yes**	**NA**	**Yes**	**Yes**	**Yes**	**7**
Damalapati et al., 2023	**Yes**	**Yes**	**Yes**	**Yes**	**NA**	**Yes**	**Yes**	**Yes**	**7**
Ramkumarsingh Tomar et al., 2022	**Yes**	**Yes**	**Yes**	**Yes**	**NA**	**Yes**	**Yes**	**Yes**	**7**
Majumder et al., 2022	**Yes**	**Yes**	**Yes**	**Yes**	**NA**	**Yes**	**Yes**	**Yes**	**7**
Ghosh et al., 2022	**Yes**	**Yes**	**Yes**	**Yes**	**NA**	**Yes**	**Yes**	**Yes**	**7**
	**Yes**	**Yes**	**Yes**	**Yes**	**NA**	**Yes**	**Yes**	**Yes**	**7**
	**Yes**	**Yes**	**Yes**	**Yes**	**NA**	**Yes**	**Yes**	**Yes**	**7**
Ghosh et al., 2022	**Yes**	**Yes**	**Yes**	**Yes**	**NA**	**Yes**	**Yes**	**Yes**	**7**
Garg & Dhamija, 2022	**Yes**	**Yes**	**Yes**	**Yes**	**NA**	**Yes**	**Yes**	**Yes**	**7**
Bhandari et al., 2022	**Yes**	**Yes**	**Yes**	**Yes**	**NA**	**Yes**	**Yes**	**Yes**	**7**
Venkatesh et al., 2021	**Yes**	**Yes**	**Yes**	**Yes**	**NA**	**Yes**	**Yes**	**Yes**	**7**
Ninama et al., 2021	**Yes**	**Yes**	**Yes**	**Yes**	**NA**	**Yes**	**Yes**	**Yes**	**7**
Soundararajan et al., 2020	**Yes**	**Yes**	**Yes**	**Yes**	**NA**	**Yes**	**Yes**	**Yes**	**7**
Saxena et al., 2020	**Yes**	**Yes**	**Yes**	**Yes**	**NA**	**Yes**	**Yes**	**Yes**	**7**

	**Yes**	**Yes**	**Yes**	**Yes**	**NA**	**Yes**	**Yes**	**Yes**	**7**
Sardana & Shringi, 2020	**Yes**	**Yes**	**Yes**	**Yes**	**NA**	**Yes**	**Yes**	**Yes**	**7**
Kaiser et al., 2020	**Yes**	**Yes**	**Yes**	**Yes**	**NA**	**Yes**	**Yes**	**Yes**	**7**
Gupta et al., 2020	**Yes**	**Yes**	**Yes**	**Yes**	**NA**	**Yes**	**Yes**	**Yes**	**7**
Garg & Dhamija, 2020	**Yes**	**Yes**	**Yes**	**Yes**	**NA**	**Yes**	**Yes**	**Yes**	**7**
Kasinathan et al., 2019	**Yes**	**Yes**	**Yes**	**Yes**	**NA**	**Yes**	**Yes**	**Yes**	**7**
Himral et al., 2019	**Yes**	**Yes**	**Yes**	**Yes**	**NA**	**Yes**	**Yes**	**Yes**	**7**
Kamalasanan CG et al., 2019	**Yes**	**Yes**	**Yes**	**Yes**	**NA**	**Yes**	**Yes**	**Yes**	**7**
Nandi & Maity, 2018	**Yes**	**Yes**	**Yes**	**Yes**	**NA**	**Yes**	**Yes**	**Yes**	**7**
Thakur et al., 2017	**Yes**	**Yes**	**Yes**	**Yes**	**NA**	**Yes**	**Yes**	**Yes**	**7**
Sharma et al., 2017	**Yes**	**Yes**	**Yes**	**Yes**	**NA**	**Yes**	**Yes**	**Yes**	**7**
Sahu et al., 2017	**Yes**	**Yes**	**Yes**	**Yes**	**NA**	**Yes**	**Yes**	**Yes**	**7**
Rajesekar et al., 2017	**Yes**	**Yes**	**Yes**	**Yes**	**NA**	**Yes**	**Yes**	**Yes**	**7**
Didel et al., 2017	**Yes**	**Yes**	**Yes**	**Yes**	**NA**	**Yes**	**Yes**	**Yes**	**7**
Sahu et al., 2017	**Yes**	**Yes**	**Yes**	**Yes**	**NA**	**Yes**	**Yes**	**Yes**	**7**
Mahajan et al., 2016	**Yes**	**Yes**	**Yes**	**Yes**	**NA**	**Yes**	**Yes**	**Yes**	**7**
Bhoil et al., 2016	**Yes**	**Yes**	**Yes**	**Yes**	**NA**	**Yes**	**Yes**	**Yes**	**7**
Premaratna et al., 2015	**Yes**	**Yes**	**Yes**	**Yes**	**NA**	**Yes**	**Yes**	**Yes**	**7**
Koti et al., 2015	**Yes**	**Yes**	**Yes**	**Yes**	**NA**	**Yes**	**Yes**	**Yes**	**7**

Kim et al., 2015	**Yes**	**Yes**	**Yes**	**Yes**	**NA**	**Yes**	**Yes**	**Yes**	**7**
Kim et al., 2015	**Yes**	**Yes**	**Yes**	**Yes**	**NA**	**Yes**	**Yes**	**Yes**	**7**
Bhat et al., 2015	**Yes**	**Yes**	**Yes**	**Yes**	**NA**	**Yes**	**Yes**	**Yes**	**7**
Karanth et al 2013	**Yes**	**Yes**	**Yes**	**Yes**	**NA**	**Yes**	**Yes**	**Yes**	**7**
Chiou et al 2013	**Yes**	**Yes**	**Yes**	**Yes**	**NA**	**Yes**	**Yes**	**Yes**	**7**
Nam et al 2010	**Yes**	**Yes**	**Yes**	**Yes**	**NA**	**Yes**	**Yes**	**Yes**	**7**
	**Yes**	**Yes**	**Yes**	**Yes**	**NA**	**Yes**	**Yes**	**Yes**	**7**

All 55 cases were of good quality.

Domains Leading explanatory questions
Selection 1. Does the patient(s) represent(s) the whole experience of the investigator or is the selection method unclear to the extent that other patients with similar presentation may not have been reported?

Ascertainment 2. Was the exposure adequately ascertained?

3. Was the outcome adequately ascertained?
Causality
4. Were other alternative causes that may explain the observation ruled out?
5. Was there a challenge/re-challenge phenomenon?
6. Was there a dose–response effect?
7. Was follow-up long enough for outcomes to occur?

Reporting
8. Is the case(s) described with sufficient details to allow other investigators to replicate the research or to allow practitioners make inferences related to their own practice?
