# Supplementary material for: Movement Disorders in Scrub Typhus: A Systematic Review
Source: Tremor Other Hyperkinet Mov (N Y). 2026 Mar 31;16:22. doi: 10.5334/tohm.1148 (PMC13045785; doi:10.5334/tohm.1148)
Supplement: Supplementary Table 1. — Tables 1a–1c. [file tohm-16-1-1148-s3.pdf]

Supplementary Table 1a: Demographic and Clinical Profile of Patients with Movement Disorders in Scrub Typhus (n = 55)

Reference (Author / Year)	Country	Age / Sex	Diagnostic Confirmation (Serology / PCR)	Systemic Features/ other system involvement	A characteristic eschar	Duration of Illness Before Neurological Onset	Onset Timing (Acute / Post-infectious)
Salini et al., 2025 (two out of five had movement disorders)	India	42/M	IgM ELISA	Fever, myalgia, mask-like facies, rigidity, tremor, mild thrombocytopenia, transaminitis	Yes (left groin)	15 days	Acute
		38/F	IgM ELISA	Fever, headache, myalgia, transaminitis, thrombocytopenia	No	10 days	Post-infectious (2nd week)
Saibaba et al., 2025	India	32/F	IgM ELISA positive for O. tsutsugamushi	Fever, altered sensorium, transaminitis, acute kidney injury, myocarditis	Yes (arm)	3 days	Acute
Biswas et al., 2025	India	23/F	Serum IgM ELISA + Weil-Felix + Immunochromatography	Fever, headache, vomiting, behavioral changes, transaminitis	No	5 days	Acute
Bahadur et al., 2025	India	12/F	Serum IgM for O. tsutsugamushi (35 U; normal <9)	High-grade fever, petechiae, thrombocytopenia	Not mentioned	Fever for 3 days → neurological onset 5 days later	Post-infectious
Alam et al., 2025	India	Preschool/M	Serum IgM ELISA positive for O. tsutsugamushi	Low-grade fever, irritability, excessive crying	No	20 days	Subacute
Thomas et al., 2024	India	11/M	NCS: AMAN variant of GBS; Serum IgM positive for O. tsutsugamushi	Fever 18 days before, transient bladder retention, swallowing difficulty	Not mentioned	~8 days after fever	Acute
Singh & Singh, 2024	India	18/F	Serum IgM ELISA positive for O. tsutsugamushi (OD 25.48)	Fever, headache, vomiting, seizures, mild pancreatitis, ascites, splenomegaly, leptomeningitis	No	10 days	Acute
Reddy et al., 2024	India	20/F	Serum IgM ELISA positive for O. tsutsugamushi (OD 2.158; cutoff 0.468)	Fever (5 days), altered sensorium (2 days), seizures (1 day), icterus	No	5 days	Acute

Puppala et al., 2024	India	14/M	Serum IgM ELISA positive for O. tsutsugamushi (titer 3.4)	Fever, vomiting, diarrhea	No	~3 days	Post-infectious
		30/M	Serum IgM ELISA positive for O. tsutsugamushi (titers 3.1 and 3.4 one week apart)	Fever, generalized tonic-clonic seizures, urinary incontinence	No	~5 days	Post-infectious
Neela et al., 2024	India	34/M	Serum IgM ELISA positive for O. tsutsugamushi (1:3200)	Fever (10 days), headache, myalgia, blurred vision	No	10 days	Acute
Meena et al., 2024	India	36/M	Serum IgM ELISA positive for O. tsutsugamushi	Fever (7 days), abdominal pain, vomiting	Yes – typical 'cigarette burn' eschar on chest	10 days (developed opsoclonus on day 3 of hospitalisation)	Acute
Li et al., 2024	China	51/F	Next-generation sequencing of blood positive for O. tsutsugamushi	Fever (1 month), gastrointestinal bleeding, confusion	Yes – 0.8 × 0.8 cm eschar in right groin	~30 days	Subacute
Jafri et al., 2024	India	60/M	Serology positive for O. tsutsugamushi	Fever (7 days), headache, arthralgia	No	10 days (hypoactive delirium on day 3 of hospitalization)	Acute
Datta et al., 2024	India	7/M	Serum and CSF IgM ELISA positive for O. tsutsugamushi	Fever, respiratory distress, initial lower limb weakness progressing to spastic quadriparesis and respiratory failure	Not specified	Few days after fever onset	Acute (rhombencephalitis) → Post-infectious (HOD)
Damalapati et al., 2023	India	35/M	Serum IgM ELISA positive for O. tsutsugamushi	Fever, vomiting, diarrhea, hypotension, transaminitis	Healing maculopapular rash (likely eschar)	~7–8 days	Acute

					on posterior neck		
Ramkumarsingh Tomar et al., 2022	India	28/F	Weil–Felix OX-K 1:320; IgM ELISA OD 1.18 positive for O. tsutsugamushi	Fever (10 days), irritability, imbalance, recurrent falls	Not reported	6 days (fever resolved in 4 days, OMS began 2 days later)	Post-infectious
Majumder et al., 2022	India	7/M	Serum and CSF positive for O. tsutsugamushi	Fever, respiratory distress	Not specified	Within a few days of fever onset	Acute
Ghosh et al., 2022 (Three out of ten had movement disorders)	India	62/M	IgM ELISA and Weil–Felix positive	Fever, myalgia, headache, jaundice	Present (shoulder)	~3 weeks	Acute
		28/F	IgM ELISA and Weil–Felix positive	Fever, headache, papilledema, positive Brudzinski's sign	Present (intergluteal cleft)	10 days	Acute
		40/F	IgM ELISA positive	Fever, headache	Absent	10 days	Acute
Ghosh et al., 2022	India	45/M	Paired serum and CSF positive for O. tsutsugamushi PCR	Fever (5 days), headache	Absent	5 days	Acute
Garg & Dhamija, 2022	India	23/F<br><br>(pregnant, 36 weeks)	IgM ELISA positive for O. tsutsugamushi	Fever (1 week), hypotension, bilateral basal crepitations, multiorgan dysfunction (renal and hepatic involvement)	Yes – submammary region	2 days (OMS appeared almost simultaneously with fever onset)	Acute
Bhandari et al., 2022	Nepal	14/M	IgM rapid antibody positive	Fever (6 days), headache (5 days), vomiting, lymphadenopathy, papilledema	Absent	~6 days	Acute
Venkatesh et al., 2021	India	18/M	Scrub IgM positive (ImmuneMed Rapid)	Fever (8 days), petechial rash (face → trunk & limbs), vomiting, transaminitis	Not reported	7 days (neurological signs appeared after 1 week of fever)	Acute
Ninama et al., 2021	India	15/M	Serum IgM ELISA positive for O. tsutsugamushi	Fever (5 days), difficulty walking (2 days), slurred speech (1 day), drowsiness, no rash, no seizures	Present (right thigh)	3 days after fever onset	Acute

Soundararajan et al., 2020	India	50/M	Serum IgM positive for O. tsutsugamushi	Fever (15 days), cough, breathlessness, hypotension, hepatomegaly	Not reporte d	15 days (1 day before admission)	Acute
Saxena et al., 2020 (Two out seven had movement disorders)	India	28/F	IgM ELISA positive	Fever (7 days), vomiting, headache, mild hepatomegaly	Not mention ed	7 days	Acute
		45/M	IgM ELISA positive	Fever (10 days), headache, vomiting	Present	10 days	Acute
Sardana & Shringi, 2020 (One out of five had ataxia)	India	32/F	IgM ELISA positive	Fever (7 days), vomiting, mild hepatosplenomegaly	Not reporte d	7 days	Acute
Kaiser et al., 2020	India	7/F	Serum and CSF IgM positive for O. tsutsugamushi	Fever (15 days), headache, vomiting, diplopia, hypertension	Absent	11 days	Acute
Gupta et al., 2020	India	26/F	IgM ELISA positive for O. tsutsugamushi	Fever, vomiting, transaminitis	Absent	4 days	Acute
Garg & Dhamija, 2020	India	NA	Not reported	Not reported	Not reporte d	Not reported	Acute (~11 days from onset in cited cases)
Kasinathan et al., 2019	India	9/M	IgM ELISA positive for O. tsutsugamushi	Fever (subacute), hepatosplenomegaly	Present (scrotal eschar)	~7 days	Acute
Himral et al., 2019	India	23/F	Weil-Felix positive (OXK)	Fever, vomiting, headache, altered sensorium	Present	~10 days	Acute
Kamalasanan CG et al., 2019	India	70/F	Positive IgM ELISA for O. tsutsugamushi	Fever, myalgia, cough, breathlessness, ARDS, myocarditis, oliguria	No	~4 days (parkinsonism appeared on day 4 of illness)	Acute
Nandi & Maity, 2018	India	3/M	IgM scrub typhus positive (IFA)	Fever, irritability, hepatomegaly	Not reporte d	12 days	Acute
Thakur et al., 2017 (one of four cases had movement disorder)	India	40/M	IgM ELISA positive for O. tsutsugamushi	Fever, vomiting, altered behavior, AKI (creatinine ↑3.1 mg/dL), transaminitis	Absent	4 days	Acute
Sharma et al., 2017	India	3/F	IgM ELISA positive (2.377; cutoff 0.5), Weil-Felix OXK 1:320	Fever (5 days), irritability, photophobia, hepatosplenomegaly	Not reporte d	3 days after fever onset	Acute

Sahu et al., 2017	India	60/M	IgM positive + IFA 1:128	Fever (2 weeks), jaundice, ARDS, renal dysfunction	Present (axillary)	~14 days	Acute
Rajesekar et al., 2017	India	Young adult / M	IgM ELISA positive (O. tsutsugamushi)	Fever, vomiting, abdominal pain, generalized lymphadenopathy, episcleritis	Present (right inguinal)	~7 days	Acute
Didel et al., 2017	India	9/M	IgM ELISA positive (OD 1.014; cutoff >0.5) and real-time PCR positive	Fever, headache, vomiting, icterus, coagulopathy, elevated transaminases	Absent	~4 days	Acute
Sahu et al., 2017	India	60/M	IgM positive (BacT/ALERT FA PLUS) and IFA 1:128	Fever with chills (2 weeks), jaundice, ARDS, renal dysfunction	Present (anterior right axilla)	~14 days	Acute
Mahajan et al., 2016	India	22/F	IgM ELISA positive (InBios)	Fever (12 days), headache, vomiting, jaundice, hepatosplenomegaly	Absent	~12 days	Acute
Bhoil et al., 2016	India	21/M	Weil-Felix test (OX-K 1:320) and IgM ELISA positive	Fever (5 days), rash	Present (scrotal scar)	5 days	Acute
Premaratna et al., 2015	Sri Lanka	62/M	IFA-IgM and IgG positive for O. tsutsugamushi Karp antigen (1:1024 → 1:16,384)	Fever, chills, malaise, myalgia, arthralgia	Present (abdominal eschar)	~5 days	Acute
Koti et al., 2015	India	26/M	Weil-Felix OX-K 1:640 and IgM positive	Fever (5 days), breathlessness (1 day)	Not reported	~5 days	Acute
Kim et al., 2015	South Korea	53/F	PCR positive for O. tsutsugamushi	Headache, fever, myalgia (3 weeks)	Present (external auditory canal)	3 weeks	Acute
Kim et al., 2015	South Korea	73/M	Indirect immunofluorescence assay: IgM 1:1280 (positive)	Tremor, fever, myalgia (5 days), mild chills	Absent	5 days	Acute
Bhat et al., 2015	India	6/F	Weil-Felix OX-K positive (1:320)	Fever (5 days), cervical lymphadenopathy	Not reported	5 days	Acute
Karanth SS,	India	24/M	Weil-Felix (OXK 1:640);	Fever, headache,	Yes –	12 days	Acute

Gupta A, Prabhu M. 2013			IgM 1:5120, IgG 1:10240 by IFA	drowsiness, elevated liver enzymes, elevated CK	right thigh		
Chiou YH, Yang CJ, Lai TH. 2013	Taiwan	55/M	Positive serum IgM for O. tsutsugamushi by IFA (1:160)	Fever, chills, mild headache, malaise, erythematous rash on trunk and limbs	No	~5 days after fever onset	Acute
Nam et al 2010	South Korea	64 / F	Serology positive for Orientia tsutsugamushi (antibody titer 1:2,560)	Drowsiness; mild nuchal rigidity; no cranial nerve dysfunction, limb weakness, or sensory loss reported	Not reported	Not reported	Acute (during infection)
		40 / M	Serology positive for Orientia tsutsugamushi (antibody titer 1:2,560)	Drowsiness; mild nuchal rigidity; no cranial nerve dysfunction, limb weakness, or sensory loss reported	Not reported	Not reported	Acute (during infection)

AKI – Acute Kidney Injury; AMAN – Acute Motor Axonal Neuropathy; ARDS – Acute Respiratory Distress Syndrome; CK – Creatine Kinase; CSF – Cerebrospinal Fluid; ELISA – Enzyme-Linked Immunosorbent Assay; F – Female; GBS – Guillain–Barré Syndrome; HOD – Hypertrophic Olivary Degeneration; IFA – Indirect Immunofluorescence Assay; IgG – Immunoglobulin G; IgM – Immunoglobulin M; M – Male; NCS – Nerve Conduction Study; OD – Optical Density; PCR – Polymerase Chain Reaction

Supplementary Table 1b: Neurological Manifestations and Laboratory Findings in Scrub Typhus–Associated Movement Disorders (n = 55)

Reference (Author / Year)	Type of Movement Disorder	Associated Neurological Findings	CSF Findings	Hematological abnormalities, if any	MRI / CT Findings
Salini et al., 2025 (two out of five had movement disorders)	Transient Parkinsonism	Bradykinesia, tremor, rigidity	Mild lymphocytic pleocytosis (15 cells/µL), normal protein & glucose	Mild thrombocytopenia, elevated SGOT/SGPT	Normal
	Opsoclonus myoclonus	Head tremor, spontaneous multidirectional saccades, ataxia	Lymphocytes (0-1 cells/µL), protein 78 mg/dL, glucose 51 mg/dL	Thrombocytopenia, transaminitis	Normal
Saibaba et al., 2025	Opsoclonus myoclonus (saccadomania)	Generalized jerky movements, multidirectional rapid saccades	10 cells/µL (lymphocytic), protein 54 mg/dL, glucose 40 mg/dL (serum 80 mg/dL)	Transaminitis, AKI, myocarditis	Normal
Biswas et al., 2025	Cortical multifocal myoclonus	Generalized convulsive status epilepticus, altered sensorium, papilledema, behavioral abnormalities	Opening pressure 30 cm H_2O; 64/µL lymphocytes; protein 84 mg/dL; glucose 103.8 mg/dL	Lymphocytic leukocytosis, elevated ESR, mild transaminitis	Bilateral basal ganglia, left parieto-temporal and bifrontal cortical hyperintensities; leptomeningeal enhancement
Bahadur et al., 2025	Opsoclonus-myoclonus-ataxia syndrome	Tremor, startle, incoordination	Normal cell counts, protein, glucose, ADA, gram stain, culture, HSV-1, VZV, JEV PCR	Thrombocytopenia	Normal MRI and EEG
Alam et al., 2025	Ballismus (hyperkinetic, violent, involuntary, high-amplitude flinging movements)	Mutism, altered sensorium, pyramidal and extrapyramidal signs	Normal CSF microscopy; negative CSF anti-NMDAR and neurotropic virus panel	Mild pallor	Bilateral symmetrical basal ganglia hyperintensities on T2, FLAIR, and DWI
Thomas et al., 2024	Dystonia<br><br>Opisthotonus	Quadriparesis, bilateral facial palsy, diminished gag reflex, meningeal signs	5 cells (lymphocytes), protein 158 mg/dL, glucose 72 mg/dL (blood glucose 120 mg/dL), albuminocytologic dissociation	None reported	Not performed
Singh & Singh, 2024	Opsoclonus–myoclonus	Generalized tonic-clonic seizures, nuchal rigidity, toxic appearance, drowsiness but	10 WBC/mm³, protein 150 mg/dL, glucose 120 mmol/L	Leukocytosis, mild anemia, elevated CRP (34.1 mg/L), ESR 47 mm/h, raised ALP (420	Leptomeningeal enhancement of bilateral cerebral hemispheres

		arousable		U/L)	
Reddy et al., 2024	Opsoclonus	Altered mental status, GCS 11/15, no focal deficits, no neck rigidity	Not performed (may show albuminocytologic dissociation)	Leukocytosis (16,000/µL), thrombocytopenia (34,000/µL), hyperbilirubinemia (7.7 mg/dL), elevated AST (215 IU/L), ALT (168 IU/L), ALP (503 IU/L)	Normal
Puppala et al., 2024	Opsoclonus, multifocal myoclonus, cerebellar ataxia	Wide-based gait, clumsiness, tendency to fall, jerky movements, no motor/sensory/autonomic involvement	Pleocytosis: 15 WBC/µL (95% lymphocytes)	Normal hematology, renal and liver functions	MRI brain: Normal
	Multifocal myoclonus, intentional tremor	Generalized rigidity (MDS-UPDRS Grade II), tremulous voice, post-ictal confusion, no cerebellar or sensory signs	CSF: Normal	Hemogram, renal, liver functions: Normal	MRI brain: Normal
Neela et al., 2024	Opsoclonus (multiaxial, involuntary saccadic eye movements)	No cranial nerve involvement, normal sensorimotor and cerebellar exam	Mild lymphocytic pleocytosis (11 cells/mm³), protein 71 mg/dL, glucose 47 mg/dL	Lymphocytosis, normal Hb, platelets, renal and hepatic function	Normal CT and MRI brain
Meena et al., 2024	Opsoclonus (chaotic, multidirectional eye movements)	No cerebellar signs, normal neurological examination except opsoclonus	Not done	Thrombocytopenia (40,000/µL), ESR 91 mm/h, hyperbilirubinemia (3.5 mg/dL), elevated AST (133 U/L), ALP (388 U/L), urea (106 mg/dL), creatinine (1.3 mg/dL)	Gliosis and encephalomalacia (sequelae of old traumatic brain injury); cerebellum normal
Li et al., 2024	Akinetic mutism	Akinetic mutism, negative Babinski, no neck rigidity	Mildly increased protein, normal glucose and chloride, lymphocytes 200/µL	Not reported	MRI: Multiple lesions in thalamus and brainstem; CT (1 month later): bilateral diffuse low-density periventricular and thalamic lesions
Jafri et al., 2024	Akinetic mutism	Mutism, apathy,	Normal (repeat LP: normal cells, protein, glucose)	Mildly elevated SGOT	MRI: Normal (T1,

		drowsiness, disorientation, no focal neurological deficits		(123.5 U/L), SGPT (127.1 U/L), normal CBC, renal function, electrolytes	T2, FLAIR, DWI); EEG: diffuse theta/delta slowing, encephalopathic pattern
Datta et al., 2024	Cerebellar ataxia (persistent)	Acute onset ataxia, dysarthria, spastic quadriparesis, respiratory failure, later cerebellar signs	Unremarkable at follow-up	Not reported	Initial MRI: diffuse medullary T2/FLAIR hyperintensity; Follow-up MRI (2 yrs): bilateral inferior olivary hyperintensity and enlargement (HOD), no contrast enhancement or diffusion restriction
Damalapati et al., 2023	Cerebellar ataxia with dysmetria, dysdiadochokinesia, and intention tremor	Cerebellar-type dysarthria, pan-directional nystagmus, truncal and limb ataxia	Lymphocytic pleocytosis (8 cells/µL), protein 64.5 mg/dL, few RBCs, normal glucose	Mild thrombocytopenia (drop from 404,000 to 168,000/µL), elevated AST (90 IU/L) and ALT (213 IU/L)	MRI: Normal
Ramkumarsingh Tomar et al., 2022	Opsoclonus, myoclonus	Drowsiness (GCS 12/15), chaotic multidirectional conjugate eye movements, jerky limb movements	45 cells/µL (100% lymphocytes), protein 999 mg/L, glucose 2.83 mmol/L, GeneXpert and BioFire negative	Leukocytosis (16 × 10^9/L), thrombocytopenia (90 × 10^9/L), elevated transaminases	MRI: Normal
Majumder et al., 2022	Acute cerebellar syndrome	Acute cerebellar signs, quadriparesis, hypertonia, exaggerated reflexes, bilateral extensor plantar, respiratory failure requiring mechanical ventilation	Normal CSF	Lymphocytic leucocytosis	MRI (Day 10): T2/FLAIR hyperintense lesion in medulla suggesting low-grade glioma vs demyelination; MRS supported demyelination; Repeat MRI (3 months): small residual lesion

Ghosh et al., 2022 (Three out of ten had movement disorders)	Parkinsonism (rest tremor, rigidity, bradykinesia, masked facies)	Hypophonic speech, obsessive behaviour	Lymphocytic pleocytosis (21 cells), protein 68 mg/dL, glucose 23 mg/dL	Anemia, leukopenia, thrombocytopenia, raised ESR, elevated transaminases	MRI normal
	Cerebellar ataxia with intention tremor, nystagmus, dysdiadochokinesia, rebound phenomenon	Ataxic speech, square wave jerks, hypotonia, impaired coordination	CSF: lymphocytic pleocytosis (45 cells), high protein (80 mg/dL), low glucose (30 mg/dL)	Neutrophilic leukocytosis, thrombocytopenia, elevated ESR	MRI: bilateral cerebellar involvement with uniform contrast enhancement
	Isolated opsoclonus (multidirectional chaotic saccades)	Oscillopsia, impaired fixation, no myoclonus or ataxia	Mild CSF protein elevation (88 mg/dL), low glucose (29 mg/dL), 3 cells	Mild thrombocytopenia, raised transaminases, mild hyponatremia	MRI: Normal
Ghosh et al., 2022	Diaphragmatic myoclonus (involuntary, rapid, arrhythmic jerks involving shoulder girdle and abdominal wall); action myoclonus and startle responses	No opsoclonus; normal higher mental functions; no cranial nerve involvement; no pelvic or lower limb involvement	Protein: 160 mg/dL; lymphocytic pleocytosis (67 cells, all lymphocytes); glucose: 30 mg/dL	Neutrophilic leukocytosis, raised ESR, CRP, mild transaminitis	MRI brain and spinal cord: normal; EEG: normal
Garg & Dhamija, 2022	Opsoclonus (chaotic, multidirectional conjugate eye movements) and generalized myoclonus	Normal tone, reflexes, no parkinsonism or cerebellar signs; normal finger–nose and tandem gait tests	Normal	Leukocytosis (13,000/mm³), thrombocytopenia (90,000/mm³), elevated BUN (64 mg/dL), creatinine (1.4 mg/dL), elevated bilirubin (2.0 mg/dL), AST (111 IU/L), ALT (121 IU/L)	MRI brain normal, CSF normal, EEG normal, PET-CT negative for malignancy
Bhandari et al., 2022	Cerebellar involvement (bilateral gaze-evoked nystagmus, ataxia, dysmetria)	Meningeal signs (neck rigidity, positive Kernig's and Brudzinski's signs); agitation	LP not performed due to raised ICP	Thrombocytopenia (122,000/µL), hyponatremia (128 mmol/L), CRP elevated (37 mg/L)	CT head: normal
Venkatesh et al., 2021	Cerebellar ataxia with broad-based gait, scanning speech, bilateral horizontal nystagmus,	Peripheral sensorimotor neuropathy (hyporeflexia, graded sensory loss up to hip), flexor plantars	Not performed (due to thrombocytopenia)	Thrombocytopenia (platelets dropped to 8×10^9/L), leukopenia, elevated AST/ALT	MRI brain: Normal

	dysmetria, dysdiadochokinesia				
Ninama et al., 2021	Cerebellar ataxia (truncal ataxia, past pointing, dysdiadochokinesia)	Neck rigidity present, Kernig's and Brudzinski's negative, no focal deficits	32 cells (70% lymphocytes), normal glucose/protein – aseptic meningitis	Platelet count 53,000, hyponatremia (Na 122), AST 152 U/L, ALT 53 U/L, albumin 2.5 g/dL	MRI brain: normal
Soundararajan et al., 2020	Parkinsonism (bilateral resting tremors, masked facies, slow hypophonic speech, bradykinesia, short-stepped gait, reduced arm swing)	Disorientation, slurred speech, insomnia, no focal deficits	Normal (5 cells, lymphocytic; protein 21.8 mg/dL; glucose 85 mg/dL)	Hyponatremia (120–130 mmol/L), hypoosmolar, increased urinary sodium (89 mmol/L), thrombocytopenia, prerenal azotemia, mild transaminitis, hypoalbuminemia	CT: parietal calcified granuloma, no acute lesion
Saxena et al., 2020 (Two out seven had movement disorders)	Cerebellar ataxia (broad-based gait, past pointing, dysdiadochokinesia)	Mild confusion, impaired tandem gait, no focal deficit	Mild lymphocytic pleocytosis, protein 78 mg/dL	Thrombocytopenia, mild hyponatremia	MRI brain: normal
	Cerebellar signs (gaze-evoked nystagmus, dysmetria, ataxia)	No focal neurological deficits, mild altered sensorium	CSF: lymphocytic pleocytosis, elevated protein	Thrombocytopenia, elevated liver enzymes	MRI: mild cerebellar hyperintensity on T2
Sardana & Shringi, 2020 (One out of five had ataxia)	Cerebellar ataxia (truncal and gait ataxia, dysmetria, intention tremor)	Mild altered sensorium, no focal deficit	CSF: lymphocytic pleocytosis, mildly raised protein	Thrombocytopenia, mild hyponatremia	MRI brain: normal
Kaiser et al., 2020	Cerebellar ataxia (unsteady gait, past pointing, dysdiadochokinesia)	Abducens palsy, neck rigidity, visual impairment due to raised ICP	102 cells/cmm (92% mononuclear), protein 119 mg/dL, glucose 59 mg/dL, scrub IgM/IgG positive	Moderate anemia, leukocytosis (15,010/cmm, neutrophilic), normal LFT/RFT	MRI brain: normal
Gupta et al., 2020	Cerebellar ataxia (broad-based gait, truncal ataxia, dysdiadochokinesia)	Horizontal gaze nystagmus, scanning speech, hypotonia	Acellular CSF, protein 35 mg/dL, glucose 50 mg/dL	Thrombocytopenia (82×10^9/L), mild transaminitis	MRI brain: normal
Garg & Dhamija, 2020	Opsoclonus (multidirectional, conjugate, involuntary saccadic eye movements – 'saccadomania')	None described (review-based)	Not reported	Not reported	Not reported
Kasinathan et al., 2019	Ocular flutter (bursts of conjugate	Cerebellar ataxia, right convergent squint	70 cells (lymphocytic), protein 105 mg/dL	Not reported	MRI brain: normal

	horizontal saccadic oscillations)				
Himral et al., 2019	Cerebellar ataxia	Multiple cranial nerve palsies (III, VI, VII), meningitis	22 cells/mm³ (lymphocytic), protein 108 mg/dL, glucose 60 mg/dL	Leukocytosis	MRI: hyperintense lesions in bilateral cerebellar hemispheres
Kamalasanan CG et al., 2019	Parkinsonism (masked facies, rigidity, resting tremor, cogwheel rigidity)	Amnesia for entire illness period (including admission and hospitalization); normal higher functions otherwise	CSF: 4 cells (100% lymphocytes), protein 15 mg/dL, sugar 69 mg/dL, ADA 5.2 (<10)	Not reported	CT: Deep white matter ischemia, age-related atrophy; MRI: Age-related atrophy
Nandi & Maity, 2018	Opsoclonus–myoclonus	None noted beyond OMS	20 cells/mm³ (95% lymphocytes), protein 35 mg/dL, glucose 92 mg/dL	Not reported	MRI brain: normal
Thakur et al., 2017 (one of four cases had movement disorder)	Opsoclonus–myoclonus	Terminal neck rigidity, altered sensorium	1–2 cells/mm³, glucose 56 mg/dL, protein 121 mg/dL	Leukocytosis (22,500/mm³), thrombocytopenia (2.1 L/mm³), mild transaminitis	MRI: Normal
Sharma et al., 2017	Cerebellar ataxia (truncal + limb ataxia, horizontal nystagmus, dysarthria)	Signs of meningeal irritation, mild photophobia	10 cells/mm³ (100% lymphocytes), normal glucose and protein	Anemia (Hb 9.7 g/dL), CRP 45 mg/dL, hypoalbuminemia (2.5 g/dL), hyponatremia (130 mEq/L), elevated AST (204 U/L), ALT (164 U/L)	MRI: Bilateral cerebellar cortical hyperintensities with folial effacement (acute cerebellitis)
Sahu et al., 2017	Opsoclonus (multiaxial, involuntary, saccadic eye movements)	None	Not reported	Thrombocytopenia (22,000/mm³), elevated bilirubin (17.3 mg/dL), deranged LFTs, elevated urea/creatinine	Chest X-ray: diffuse opacities (ARDS); no brain imaging reported
Rajesekar et al., 2017	Opsoclonus, choreiform movements, cogwheel rigidity	Left UMN facial palsy, pyramidal signs, cerebellar signs (ataxia, dysmetria, dysdiadochokinesia, hypotonia)	Protein 146.3 mg/dL, glucose 45 mg/dL, chloride 114 mEq/L, 50 cells/mm³	Leukocytosis (20,100), lymphocytosis (70%), Monocytosis (11%), thrombocytopenia (1.94 lakh/mm³), elevated AST/ALT, LDH 598	Chest X-ray: increased bronchovesicular markings (no MRI reported)
Didel et al., 2017	Cerebellar ataxia (left truncal ataxia, horizontal gaze nystagmus)	None reported	Acellular CSF, glucose 94 mg/dL, protein 56 mg/dL	Leukocytosis (12.2 ×10⁹/L), lymphocytosis (88%), thrombocytopenia (76 ×10⁹/L),	MRI: focal T2 hyperintensity in left cerebellar tonsil

				hyperbilirubinemia, elevated AST/ALT	
Sahu et al., 2017	Opsoclonus (multiaxial, involuntary saccadic eye movements)	Drowsiness, GCS 12/15	Not reported	Thrombocytopenia (22,000/mm³), elevated bilirubin (17.3 mg/dL), AST/ALT ↑, ALP ↑, serum urea 132 mg/dL, creatinine 2.6 mg/dL	CXR: bilateral diffuse opacities (ARDS); no neuroimaging reported
Mahajan et al., 2016	Cerebellar ataxia (truncal and gait), square wave jerks, scanning speech	Hypotonia, rebound phenomenon, dysdiadochokinesia, impaired finger–nose and knee–heel test	15 lymphocytes/mm³, protein 90 mg/dL, glucose 52 mg/dL, ADA 2.0 IU/L	Leukocytosis (13,420/mm³), elevated bilirubin (6.4 mg/dL), AST 249 IU/L, ALT 217 IU/L, ALP 495 IU/L, hypoalbuminemia (2.5 g/dL)	MRI: uniform pachymeningeal enhancement with bilateral cerebellar edema
Bhoil et al., 2016	Cerebellar ataxia, severe truncal and gait ataxia, slurred speech	Semiconscious state	Normal	Not reported	MRI: diffuse cerebellar cortical hyperintensity on T2 and FLAIR, diffusion restriction and post-contrast enhancement
Premaratna et al., 2015	Parkinsonism (intermittent resting tremor of right arm and leg, rigidity, mask-like face)	None	Not performed	Lymphocytic leukocytosis, ESR 80 mm/h	CT brain and EEG: normal
Koti et al., 2015	Opsoclonus–myoclonus (saccadomania, myoclonic jerks in limbs, head titubation)	Increased tone, hyperreflexia	Normal (no meningoencephalitis)	Leukocytosis (14,300/mm³) with lymphocytosis (74%); elevated ALT (394), AST (145)	MRI: no structural lesion
Kim et al., 2015	Cerebellar ataxia (ataxic leg movement), slurred speech	Lateral gaze palsy, abnormal cerebellar function tests, gait untestable due to severity	Protein 514.8 mg/dL, glucose 42 mg/dL, WBC 80,000/mm³ (92% lymphocytes), RBC 520/mm³, culture negative	CRP 7.32 mg/dL (mildly elevated)	MRI: acute intracranial hemorrhage, SAH, enhancing lesion in right cerebellum, pons, and left midbrain. Follow-up CT: new SAH, IVH,

					lesion progression
Kim et al., 2015	Parkinsonism (resting & postural tremor, rigidity, bradykinesia, mask-like facies)	Reduced arm swing, mild gait slowness, hyperreflexia	Not performed	Thrombocytopenia (79,000/mm³), CRP 8.61 mg/dL, ferritin 5788.08 ng/mL, elevated AST/ALT	Mild diffuse cerebral and medial temporal lobe atrophy; chronic lacunes in left basal ganglia & right cerebellum
Bhat et al., 2015	Cerebellar ataxia (scanning speech, gait disturbance)	One episode of GTCS, decreased tone, power 4/5 in all limbs, no meningeal signs	Normal	ESR 25 mm/h, CRP 26.9 mg/L, WBC 10,500/mm³	CT: effacement of cerebellar folia with early hydrocephalus; MRI: diffuse cerebellar cortical hyperintensity on T2 and FLAIR, swelling without enhancement
Karanth SS, Gupta A, Prabhu M. 2013	Cerebellar ataxia (gross truncal ataxia, ataxic speech, nystagmus)	Horizontal gaze nystagmus, dysdiadochokinesia, past pointing; no cranial nerve palsy, motor or sensory deficit	Mild lymphocytic pleocytosis (25 cells/mm³), protein 60 mg/dL, cultures negative	Leukocytosis (14×10⁹/L), neutrophilia (56%), thrombocytopenia (93×10⁹/L)	MRI normal
Chiou YH, Yang CJ, Lai TH. 2013	Parkinsonism (mask-like face, bradykinesia, rigidity, small-stepped gait) and myoclonus (bilateral, arrhythmic, proximal & distal, decreased with voluntary movement)	Tremor (5–7 Hz, postural and intentional), mild slow speech, facial immobility, no stooped posture or retropulsion, intact postural reflex	Not performed (patient refused LP)	Normal hematology and biochemistry	MRI brain normal
Nam et al 2010	Opsoclonus	Drowsiness; mild nuchal rigidity	WBC 49/mm³; protein 102 mg/dL	Not reported	Brain MRI normal
	Opsoclonus	Drowsiness; mild nuchal rigidity	WBC 28/mm³; protein 91 mg/dL	Not reported	Brain MRI normal

ADA – Adenosine Deaminase; AKI – Acute Kidney Injury; ALP – Alkaline Phosphatase; ALT – Alanine Aminotransferase; AST – Aspartate Aminotransferase; CBC – Complete Blood Count; CRP – C-Reactive Protein; CSF – Cerebrospinal Fluid; CT – Computed Tomography; CXR – Chest X-ray; DWI – Diffusion-Weighted Imaging; EEG – Electroencephalogram; ESR – Erythrocyte Sedimentation Rate; FLAIR – Fluid-Attenuated Inversion Recovery; GCS – Glasgow Coma Scale; GTCS – Generalized Tonic-Clonic Seizure; Hb – Hemoglobin; HOD – Hypertrophic Olivary Degeneration; HSV – Herpes Simplex Virus; ICP – Intracranial Pressure; JEV – Japanese Encephalitis Virus; LP – Lumbar Puncture; LFT – Liver Function Test; MDS-UPDRS – Movement Disorder Society–Unified Parkinson's Disease Rating Scale; MRI – Magnetic Resonance Imaging; MRS – Magnetic Resonance Spectroscopy; NMDAR – N-Methyl-D-Aspartate Receptor; RBC – Red Blood Cells; SAH – Subarachnoid Hemorrhage; SGOT – Serum Glutamic-Oxaloacetic Transaminase; SGPT – Serum Glutamic-Pyruvic Transaminase; UMN – Upper Motor Neuron; VZV – Varicella Zoster Virus; WBC – White Blood Cells

Supplementary Table 1c: Treatment, Outcomes, and Proposed Mechanisms in Scrub Typhus–Associated Movement Disorders (n = 55)

Reference (Author / Year)	Treatment (Antibiotics, Immunotherapy, Symptomatic)	Response to Treatment	Outcome	Duration of Follow-up	Proposed Mechanism
Salini et al., 2025 (two out of five had movement disorders)	IV doxycycline	Tremor and rigidity improved by day 10, complete recovery in 2 weeks	Complete recovery	2 weeks	Cytokine-mediated endothelial activation, BBB disruption, microglial activation damaging dopaminergic neurons
	IV doxycycline + IV methylprednisolone	Eye movements resolved, mild head tremor persisted but improved	Complete recovery	2 weeks	Immune-mediated, antibody response with IgG class switch
Saibaba et al., 2025	Doxycycline 100 mg BD + Dexamethasone 6 mg q6h × 10 days	Marked improvement by day 3; complete resolution by day 10	Complete recovery	10 days (in-hospital)	Type II hypersensitivity with IgG cross-reaction damaging pontine, midbrain, and cerebellar structures; direct CNS invasion also possible
Biswas et al., 2025	IV lorazepam, phenytoin, levetiracetam, lacosamide, clobazam, ceftriaxone, vancomycin, acyclovir → shifted to oral doxycycline	Seizures stopped in 24h; regained consciousness in 48h; fever resolved in 4 days; MRI lesions resolved by 3 months	Complete recovery, seizure-free, no residual deficits	3 months	Endothelial invasion, vasculitis, macrophage activation (NF-κB/AP-1), chemokine expression (MIP-1α/β, MCP-1, IL-8), immune-mediated inflammation causing parenchymal damage
Bahadur et al., 2025	Doxycycline	Symptomatic improvement from day 3; mild residual ataxia at 1 week	Partial recovery with mild residual signs	1 week	Parainfectious immune-mediated phenomenon suggested by asymptomatic interval and normal CSF findings
Alam et al., 2025	Third-generation cephalosporin, acyclovir, oral doxycycline, IVIG (2 g/kg), IV methylprednisolone (30 mg/kg/day × 3 days) → oral steroids, clobazam	Abnormal movements and irritability resolved by day 2; regained speech by day 4; walking without support by day 5	Full recovery, no relapses	1 year	Likely immune-mediated basal ganglia encephalitis triggered by scrub typhus (possibly anti-DR2 antibodies)
Thomas et al., 2024	IVIG × 5 days, pregabalin,	Gradual improvement; opisthotonus resolved; power	Near-complete	3 months	Likely radicular involvement in GBS triggered by scrub typhus leading to imbalance between flexor–extensor

	amitriptyline, NSAIDs, gabapentin, azithromycin	recovery in 3 months	recovery with mild residual weakness		tone and antagonist–agonist muscles
Singh & Singh, 2024	Doxycycline 100 mg 12-hourly, ceftriaxone 2 g 12-hourly, levetiracetam 500 mg 12-hourly, paracetamol, supportive care	Gradual improvement; resolution of neurological and systemic signs; discharged after 11 days	Complete recovery	11 days (in-hospital)	Antigen-mediated vasculopathy with endothelial invasion leading to systemic inflammation and para-infectious OMS
Reddy et al., 2024	IV doxycycline 100 mg BID × 7 days (3 days IV, then oral); IV levetiracetam for seizures (discontinued after 4 days)	Complete resolution of opsoclonus and normalization of mental status by end of treatment	Complete recovery	7 days (in-hospital)	Immune-mediated dysfunction of pontine reticular omnipause neurons and Purkinje cells leading to disinhibition of saccadic burst neurons
Puppala et al., 2024	Oral doxycycline × 14 days	Complete resolution of opsoclonus, myoclonus, and ataxia	Full recovery, no residual deficits	3 months	Immune-mediated CNS involvement with vasculitis; likely basal ganglia and cerebellar dysfunction
	Oral doxycycline × 14 days + anti-epileptics × 3 months	Seizures resolved; myoclonus and tremor improved completely	Full neurological recovery, no residual deficits	3 months	Immune-mediated post-infectious encephalitic process with vasculitic and basal ganglia involvement
Neela et al., 2024	IV doxycycline 100 mg BID + azithromycin	Opsoclonus improved after 3 days, resolved by day 4	Complete recovery	Not specified	Breakdown of Purkinje cell inhibition → disinhibition of saccadic burst neurons in pontine reticular formation and cerebellar fastigial nucleus
Meena et al., 2024	Oral doxycycline × 10 days + supportive care	Opsoclonus resolved during hospitalization	Full recovery, discharged in stable condition	Not specified	Dysfunction of Purkinje cells in the dorsal vermis → loss of inhibition of saccadic burst neurons in the pontine reticular formation → disinhibition of cerebellar fastigial nucleus
Li et al., 2024	Doxycycline + supportive therapy	Fever improved and eschar resolved; akinetic mutism persisted	Persistent akinetic mutism	1 month	Direct CNS invasion with thalamic and brainstem involvement causing structural neuronal damage
Jafri et al., 2024	Oral doxycycline → IV doxycycline; IV	Dramatic improvement within 2 doses of modafinil (alert,	Full recovery,	Regular follow-up,	Immune-mediated neuroinflammation and endothelial dysfunction causing neuropsychiatric dysfunction;

	acyclovir, IV dexamethasone (no response); antipsychotics and benzodiazepines (no response); modafinil (dramatic response)	talkative, ambulatory)	discharged on modafinil 50 mg BID; all medications discontinued at follow-up	asymptomatic	possible modulation of dopaminergic systems (modafinil responsiveness)
Datta et al., 2024	IV azithromycin (10 mg/kg/day) + pulse methylprednisolone (30 mg/kg/day × 5 days); steroids tapered	Fever and acute deficits improved; cerebellar ataxia persisted	Residual ataxia but clinically stable at 2 years	2 years	Likely immune-mediated brainstem demyelination causing interruption of the Guillain–Mollaret triangle and secondary bilateral hypertrophic olivary degeneration
Damalapati et al., 2023	IV methylprednisolone (1 g/day × 3 days), doxycycline (100 mg twice daily × 14 days), supportive care	Afebrile and improved cerebellar signs by day 3; Barthel index improved from 0 to 55 on discharge	Significant improvement by day 30 follow-up (Barthel index 90)	30 days	Likely immune-mediated cerebellitis with vasculitic involvement rather than direct infection
Ramkumarsingh Tomar et al., 2022	IV doxycycline (7 days), oral clonazepam (up to 3 mg/day), IV immunoglobulin (2 g/kg × 5 days)	Mild initial improvement with doxycycline/clonazepam; marked improvement with IVIG; near-complete resolution in 10 days	No relapse of OMS at 1-year follow-up	1 year	Likely para-infectious autoimmune response; vasculitis and perivasculitis of endothelial cells, mononuclear infiltrates, typhus nodules, and microhaemorrhages
Majumder et al., 2022	IV methylprednisolone 30 mg/kg (pulse), IV azithromycin 10 mg/kg from Day 5, oral steroids tapered over 6 weeks	Significant neurological improvement by Day 7, extubated Day 9, improved ICP, minimal residual abnormality on discharge	Near-complete recovery with minimal residual findings	3 months	Likely immune-mediated demyelination triggered by scrub typhus infection
Ghosh et al., 2022 (Three out of ten had movement disorders)	Doxycycline (200 mg/day × 4 weeks); later pramipexole, levodopa-carbidopa, sertraline, trihexyphenidyl	Infection resolved in 2 weeks; parkinsonism persisted initially but improved by 3 months; no relapse at 1 year	Mild residual features, functionally independent	12 months	Likely immune-mediated basal ganglia involvement or vascular injury secondary to vasculitis

	Doxycycline (200 mg/day × 4 weeks) + IV dexamethasone (12 mg/day × 10 days)	Afebrile by day 6, cerebellar features improved significantly by day 10, walking unaided by week 4	Residual subtle cerebellar signs improved by 8 weeks	8 weeks	Immune-mediated cerebellitis secondary to scrub typhus
	Doxycycline (200 mg/day × 3 weeks)	Rapid improvement after 4 days, no recurrence at 6 months	Complete recovery	6 months	Para-infectious immune-mediated opsoclonus due to brainstem involvement
Ghosh et al., 2022	Doxycycline (200 mg/day) + azithromycin (600 mg/day)	Afebrile and headache reduced after 72 h; myoclonic jerks disappeared by day 8	Complete recovery	Not reported	Likely immune-mediated mechanism due to endothelial dysfunction and subcortical involvement causing phrenic nerve pathway irritation
Garg & Dhamija, 2022	Injectable azithromycin × 10 days, supportive care, emergency cesarean section	OMS resolved spontaneously over 2 weeks without specific immunotherapy	Full recovery, no relapse	Not reported	Likely parainfectious immune-mediated mechanism, possibly accelerated by pregnancy-induced rapid IgM-to-IgG switch allowing CNS penetration; transient autoantibody binding with weak neuronal injury potential
Bhandari et al., 2022	IV chloramphenicol × 14 days; IV mannitol, hypertonic saline; supportive care	Resolution of nystagmus and ataxia within days of treatment	Complete recovery	Not reported	Likely due to endothelial inflammation and CNS vasculitis leading to meningeal irritation and cerebellar involvement
Venkatesh et al., 2021	Empirical ceftriaxone + oral doxycycline (continued 2 weeks)	Rapid neurological improvement, complete resolution by discharge; normal neuro exam and LFTs by 10 days	Full recovery with no recurrence at 4 months	4 months	Likely endothelial infection with vascular injury causing cerebellar demyelination and peripheral nerve involvement
Ninama et al., 2021	Oral doxycycline 4 mg/kg/day × 7 days	Dramatic improvement within 48 h, steady gait by discharge	Complete recovery	8 days (hospital stay)	Likely due to vascular endothelial invasion by O. tsutsugamushi causing cytokine-mediated inflammation and microvascular injury leading to cerebellar involvement
Soundararajan et al., 2020	IV fluids, ceftriaxone, doxycycline × 14 days; sodium correction; blood transfusion	Afebrile within 48 h, full neurological recovery by day 8, sodium normalized (136 mmol/L) by follow-up	Complete recovery without relapse	1 week	Likely endothelial invasion and perivascular inflammation causing basal ganglia dysfunction; hyponatremia (cerebral salt wasting) contributed to encephalopathy and movement disorder
Saxena et al., 2020 (Two out seven had movement disorders)	Doxycycline × 14 days	Ataxia resolved within 1 week of treatment	Full recovery	Not reported	Likely immune-mediated cerebellar involvement following endothelial injury
	Doxycycline × 14 days + supportive care	Marked improvement within 5 days, complete resolution by 2 weeks	Full recovery	Not reported	

Sardana & Shringi, 2020 (One out of five had ataxia)	Doxycycline × 14 days	Neurological signs resolved by day 7 of treatment	Complete recovery	Not reported	Likely immune-mediated cerebellar involvement secondary to endothelial injury
Kaiser et al., 2020	Empirical ceftriaxone, hypertonic saline, then oral doxycycline (5 mg/kg/day × 7 days)	Dramatic improvement within 48 h, gait normalized, vision restored	Complete recovery, no residual deficits	8 days (hospital stay)	Likely vascular endothelial invasion with immune-mediated cerebellitis and intracranial hypertension
Gupta et al., 2020	Doxycycline 100 mg BID × 10 days, fluids, antipyretics	Afebrile by day 4; ataxia improved by day 10	Near-complete recovery at 4 weeks, minimal residual nystagmus	4 weeks	Likely vascular endothelial invasion causing cerebellar inflammation
Garg & Dhamija, 2020	Supportive care; spontaneous resolution common; steroids or IVIg sometimes used	Spontaneous resolution usually within 2–14 days	Complete recovery in most reported cases	Not reported	Likely T2-hypersensitivity–mediated autoimmune response with transient antibody binding and possible endothelial involvement
Kasinathan et al., 2019	IV doxycycline + dexamethasone × 5 days	Complete neurological recovery	Full recovery	Not reported	Likely immune-mediated cerebellitis with dysfunction of omnipause neurons in the paramedian pontine reticular formation or fastigial nucleus leading to ocular flutter
Himral et al., 2019	Doxycycline + supportive care	Gradual neurological improvement over 2 weeks	Complete recovery	Not reported	Likely immune-mediated cerebellitis with vasculitic involvement of cranial nerve nuclei and meningoencephalitic inflammation
Kamalasanan CG et al., 2019	Doxycycline, piperacillin-tazobactam, steroids, benzhexol	Rigidity resolved within 1 week; complete disappearance of extrapyramidal features; amnesia persisted for illness period	Full recovery except persistent amnesia for illness period	Follow-up duration not specified	Likely direct CNS involvement due to vasculitis/perivasculitis by O. tsutsugamushi affecting basal ganglia and memory circuits
Nandi & Maity, 2018	Oral doxycycline	Afebrile by day 5; neurological symptoms resolved by day 10	Full recovery	3 months	Likely immune-mediated parainfectious response
Thakur et al., 2017 (one of four cases had movement disorder)	IV acyclovir (empirical) + IV doxycycline	Fever resolved and consciousness normalized with doxycycline; opsoclonus persisted ~2 weeks	Full recovery	Not reported	Likely immune-mediated parainfectious opsoclonus–myoclonus with endothelial inflammation and CNS vasculitis
Sharma et al.,	IV ceftriaxone →	Fever and photophobia	Full	1 week	Likely endothelial invasion by O. tsutsugamushi causing

2017	switched to IV doxycycline (5 mg/kg/day × 10 days)	resolved within 48 h; gradual motor recovery	recovery, mild ataxia resolved by 1 week follow-up		vasculitis, cytokine release, and focal cerebellar inflammation
Sahu et al., 2017	Azithromycin + doxycycline, steroids for ARDS, NIV → Venturi mask	Opsoclonus decreased by day 2 and resolved by day 3	Full recovery	At discharge (~12 days)	Likely parainfectious immune-mediated dysfunction of burst and omnipause neurons in brainstem saccadic control circuitry
Rajesekar et al., 2017	Doxycycline 100 mg BID × 7 days	Gradual resolution of symptoms; complete recovery	Full recovery	Not reported	Likely immune-mediated demyelination (ADEM) triggered by O. tsutsugamushi with endothelial activation, cytokine release, and cross-reactive immune response
Didel et al., 2017	Initial cefotaxime, acyclovir, lactulose, rifaximin → switched to oral doxycycline	Became afebrile within 48 hours; cerebellar symptoms resolved within 1 week	Full recovery	1 week	Likely endothelial invasion with focal vasculitis and lymphocytic perivascular inflammation leading to focal cerebellar involvement
Sahu et al., 2017	Azithromycin + doxycycline, steroids for ARDS, non-invasive ventilation → Venturi mask	Opsoclonus decreased by day 2, resolved by day 3	Full recovery	~12 days (discharge)	Likely parainfectious immune-mediated dysfunction of burst and omnipause neurons in the brainstem saccadic control system
Mahajan et al., 2016	Doxycycline 100 mg BD × 14 days + IV dexamethasone 4 mg TDS × 10 days	Afebrile by day 3, able to sit by day 7, walk with support by day 9, full recovery by 4 weeks	Complete recovery	4 weeks	Likely due to endothelial vasculitis, lymphocytic inflammation, and microglial activation causing focal cerebellar injury
Bhoil et al., 2016	Oral doxycycline	Marked clinical improvement	Full recovery	Not reported	Likely due to vasculitic and immune-mediated inflammation affecting cerebellar tissue
Premaratna et al., 2015	Oral doxycycline + azithromycin	Fever resolved within 48 h, parkinsonian features resolved completely by 2 weeks	Full recovery	2 weeks	Likely immune-mediated basal ganglia involvement or inflammatory cytokine-mediated disruption of dopaminergic pathways during acute infection
Koti et al., 2015	Doxycycline for 1 week	Afebrile by day 2; opsoclonus–myoclonus resolved in 2 days	Complete recovery	2 weeks	Likely para-infectious immune-mediated dysfunction of cerebellar–brainstem circuitry
Kim et al., 2015	Doxycycline, intracranial pressure control	Rapid neurological deterioration, coma, death on day 7	Death on day 7	7 days	Severe endothelial damage and vasculitis leading to vessel rupture and intracerebral hemorrhage
Kim et al., 2015	Doxycycline 200 mg orally for 5 days	Fever and systemic markers normalized by day 5; marked motor improvement by 2 weeks (UPDRS ↓ from 21 to 2)	Complete recovery except minimal	2 weeks	Likely direct basal ganglia involvement due to vasculitis or immune-mediated mechanism triggered by O. tsutsugamushi infection

			hand tremor		
Bhat et al., 2015	Doxycycline (dose not specified)	Clinical improvement (details not reported)	Recovered (details not specified)	Not reported	Likely immune-mediated cerebellar inflammation secondary to vasculitis caused by O. tsutsugamushi
Karanth SS, Gupta A, Prabhu M. 2013	Ceftriaxone IV + doxycycline oral; doxycycline continued 14 days	Fever subsided within 3 days, neurological improvement rapid and complete	Full recovery without deficits	Not specified (short-term until discharge)	Disseminated vasculitis/perivasculitis due to endothelial invasion by O. tsutsugamushi
Chiou YH, Yang CJ, Lai TH. 2013	Oral doxycycline 100 mg BID; amantadine 100 mg BID; clonazepam 1 mg TID	Fever resolved and neurological symptoms improved gradually; myoclonus and parkinsonism improved significantly	Only fine postural tremor remained at 1 month follow-up	1 month	Direct CNS involvement with vasculitis/perivasculitis by O. tsutsugamushi; acute infection rather than post-infectious mechanism (onset 2 days after fever)
Nam et al 2010	Not reported	Not reported	Not reported	Not reported	Dysfunction of pause cells in parapontine reticular formation causing loss of tonic inhibition of saccadic burst neurons; infection-triggered mechanism suspected
	Not reported	Not reported	Not reported	Not reported	Dysfunction of pause cells in parapontine reticular formation causing loss of tonic inhibition of saccadic burst neurons; infection-triggered mechanism suspected

ADEM – Acute Disseminated Encephalomyelitis; AKI – Acute Kidney Injury; AP-1 – Activator Protein-1; ARDS – Acute Respiratory Distress Syndrome; BBB – Blood–Brain Barrier; BD – Bis in Die (twice daily); BID – Bis in Die (twice daily); BUN – Blood Urea Nitrogen; CNS – Central Nervous System; CSF – Cerebrospinal Fluid; ESR – Erythrocyte Sedimentation Rate; GBS – Guillain–Barré Syndrome; ICP – Intracranial Pressure; IgG – Immunoglobulin G; IgM – Immunoglobulin M; IL-8 – Interleukin-8; IV – Intravenous; IVIG – Intravenous Immunoglobulin; MCP-1 – Monocyte Chemoattractant Protein-1; MIP-1α/β – Macrophage Inflammatory Protein-1 alpha/beta; MRI – Magnetic Resonance Imaging; NF-κB – Nuclear Factor kappa B; NSAIDs – Non-Steroidal Anti-Inflammatory Drugs; OMS – Opsoclonus Myoclonus Syndrome; q6h – Every 6 hours; TDS – Ter Die Sumendum (three times daily); UPDRS – Unified Parkinson's Disease Rating Scale

References

1. Salini NR, Sreekumar TS, Ramakrishnan S, Srikantan S, Antony J. Rare neurological manifestations of scrub typhus: Case series from a tertiary care centre in Kerala, India. J Clin Diagn Res. 2025;19(1):OR01–OR05. doi:10.7860/JCDR/2025/72611.20533.

2. Saibaba J, Chandramouli C, Amalnath D, Subrahmanyam DKS. Scrub saccadomania: An enigma of eye movement in the tropics. J Assoc Physicians India. 2025;73(3):e39–e40. doi:10.59556/japi.73.0872.

3. Biswas U, Leon-Ruiz M, Ghosh R, Sarkar R, Bheeman R, Mukhopadhyay A, et al. Scrub typhus meningoencephalitis presenting as generalized convulsive status epilepticus with basal ganglia and extra-limbic cortical involvement, complicated by cortical multifocal myoclonus. Neurohospitalist. 2025;15(2):182–7. doi:10.1177/19418744241276903.

4. Bahadur M, Qavi A, Kulshreshtha D. Scrub typhus presenting as opsoclonus myoclonus ataxia syndrome. Neurology. 2025;105(6):e214112. doi:10.1212/WNL.0000000000214112.

5. Alam A, Verma S, Verma N, Singh D. Basal ganglia encephalitis as an atypical presentation of scrub typhus. Neurol India. 2025;73(4):925–6. doi:10.4103/0028-3886.478106.

6. Thomas J, Tandon R, Mani VE. Opisthotonic posturing in Guillain–Barre syndrome. Int J Neurosci. 2024;1–3. doi:10.1080/00207454.2024.2392120.

7. Singh K, Singh I. Unveiling the unconventional: exploring atypical manifestations of scrub typhus. Indian J Case Rep. 2024;10(10):328–9. doi:10.32677/ijcr.v10i10.4669.

8. Reddy SP, Tuli A, Yuvasai KP, Kumar Pannu AK. Opsoclonus in scrub typhus. BMJ Case Rep. 2024;17(5):e260304. doi:10.1136/bcr-2024-260304.

9. Puppala S, Acharya A, Choudhury SS. Scrub typhus with opsoclonus-myoclonus-ataxia-seizure as primary presentations. J Neurosci Rural Pract. 2024;15(1):143–7. doi:10.1055/s-0043-1776304.

10. Neela A, Gohil R, Tagore R, Ta V. Opsoclonus: A rare neurological manifestation in a patient with scrub typhus infection. Cureus. 2024;16(9):e70058. doi:10.7759/cureus.70058.

11. Meena R, Ratan G, Anuradha S, Singla S. Opsoclonus in a patient with scrub typhus infection: A rare neurological manifestation. J Indian Acad Clin Med. 2024;25(4):237–8.

12. Li H, Lai Z, Tang W. Magnetic resonance imaging features of encephalitis of scrub typhus. JAMA Neurol. 2024;81(6):654–5. doi:10.1001/jamaneurol.2024.0454.

13. Jafri AD, Dhar SK, Naik C, Rizvi K. Hypoactive delirium: A rare manifestation of scrub typhus. Cureus. 2024;16(10):e70740. doi:10.7759/cureus.70740.

14. Datta AK, Mukherjee A, Biswas A. Post-infective rhombencephalitis with bilateral olivary hypertrophic degeneration. Ann Indian Acad Neurol. 2024;27(2):210–2. doi:10.4103/aian.aian_1234_23.

15. Damalapati S, Deshpande R, Moola NS, Jadav RH, Havannavar S. Rickettsial cerebellitis: A rare neurological manifestation. Cureus. 2023;15(8):e42901. doi:10.7759/cureus.42901.

16. Ramkumarsingh Tomar L, Jatinbhai Shah D, Agarwal U, Gogia A, Rohatgi A, Agrawal CS. Scrub typhus meningoencephalitis presenting as opsoclonus myoclonus syndrome: A video-based case. Trop Doct. 2022;52(1):192–5. doi:10.1177/00494755211067355.

17. Majumder S, Samanta M, Sinha Mahapatra TK. Acute demyelination of the medulla oblongata owing to scrub typhus in a 7-year-old boy: case report. Paediatr Int Child Health. 2022;42(1):48–51. doi:10.1080/20469047.2020.1833914.

18. Ghosh R, Mandal A, León-Ruiz M, Roy D, Das S, Dubey S, et al. Rare neurological and neuropsychiatric manifestations of scrub typhus: A case series of 10 cases. Neurologia. 2022;37(9):747–54. doi:10.1016/j.nrl.2022.03.007.

19. Ghosh R, Leon-Ruiz M, Bandyopadhyay S, Roy D, Benito-Léon J. Scrub typhus presenting as diaphragmatic myoclonus. Neurol Sci. 2022;43(6):4023–4. doi:10.1007/s10072-022-06021-y.

20. Garg D, Dhamija RK. Opsoclonus-myoclonus syndrome as a heralding feature of scrub typhus: An illustrative case with a video vignette. J Mov Disord. 2022;15(1):80–2. doi:10.14802/jmd.20148.

21. Bhandari S, Bhandari S, Gautam K, Jha R, Devkota S. Meningeal signs and cerebellar involvement in scrub typhus: A case report. Cureus. 2022;14(6):e25708. doi:10.7759/cureus.25708.

22. Venkatesh MH, Viswanathan S, Selvaraj J, Pillai V. Acute cerebellar ataxia and peripheral neuropathy due to an atypical infection. BMJ Case Rep. 2021;14(3):e242229. doi:10.1136/bcr-2021-242229.

23. Ninama R, Gurjar MK, Katara LP, Jain B. Acute cerebellar ataxia: a rare presentation of scrub typhus in pediatric age group. IP Int J Med Paediatr Oncol. 2021;7(4):215–7. doi:10.18231/j.ijmpo.2021.044.

24. Soundararajan S, Viswanathan S, Jain D, Krishnamurthy V, Gayathri MS. Acute parkinsonism and cerebral salt-wasting-related hyponatremia in scrub typhus. Cureus. 2020;12(1):e6706. doi:10.7759/cureus.6706.

25. Saxena P, Chadha D, Goyal R. A case series on neurological insights of scrub typhus. J Clin Diagn Res. 2020;14(11):OR05–11. doi:10.7860/JCDR/2020/46481.14286.

26. Sardana V, Shringi P. Neurological manifestations of scrub typhus: A case series from a tertiary care hospital in southern east Rajasthan. Ann Indian Acad Neurol. 2020;23(6):808–11. doi:10.4103/aian.AIAN_301_20.

27. Kaiser RS, Khemka A, Roy O, Das S, Datta K. An unusual etiology of cerebellar ataxia. Child Neurol Open. 2020;7:2329048X20907754. doi:10.1177/2329048X20907754.

28. Gupta S, Grover S, Gupta M, Kaur D. Cerebellitis as a rare manifestation of scrub typhus fever. BMJ Case Rep. 2020;13(5):e233993. doi:10.1136/bcr-2019-233993.

29. Garg D, Dhamija R. Opsoclonus in scrub typhus. Ann Indian Acad Neurol. 2020;23(3):367. doi:10.4103/aian.AIAN_34_20.

30. Kasinathan A, Suthar R, Sahu JK, Sankhyan N, Nallasamy K. Ocular flutter in scrub typhus. J Pediatr. 2019;204:315. doi:10.1016/j.jpeds.2018.09.027.

31. Himral P, Sharma KN, Kudial S, Himral S. Scrub meningitis complicated by multiple cranial nerve palsies and cerebellitis. J Assoc Physicians India. 2019;67:88–9.

32. Kamalasanan CG, Chechattil A, Kiran K. Parkinsonism and amnesia as rare neurological manifestations of scrub typhus. *Journal of Medical Science and Clinical Research (JMSCR)*. 2019;7(7):73–6. doi:10.18535/jmscr/v7i7.15.

33. Nandi M, Maity D. Opsoclonus myoclonus syndrome: a presenting feature of scrub typhus in a child. Child Newborn. 2018;22:8–9.

34. Thakur I. Scrub typhus encephalitis: A case series from West Bengal. Bengal Physician J. 2017;6(2):33–5.

35. Sharma S. Acute cerebellar ataxia in a 3-year-old Bengali girl: a novel presentation of scrub typhus in pediatric age group. Int J Contemp Pediatr. 2017;4(2):652–4.

36. Sahu D, Varma M, Vidyasagar S. Opsoclonus in scrub typhus. J Clin Sci Res. 2017;6(2):113–6.

37. Rajesekar D, Abitha V, Ameen N. A rare presentation of scrub typhus as ADEM. J Evol Med Dent Sci. 2017;6(26):2211–3.

38. Didel S, Basha AM, Biswal M, Suthar R, Sankhyan N. Acute cerebellitis in a child with scrub typhus. *Pediatr Infect Dis J*. 2017;36(7):696–7. doi: 10.1097/INF.0000000000001532.

39. Sahu DS, Varma MV, Vidyasagar SV. Opsoclonus in scrub typhus. J Clin Sci Res. 2017;6:113–6.

40. Mahajan SK, Sharma S, Kaushik M, Raina R, Thakur P, Taneja GP, et al. Scrub Typhus Presenting as Acute Cerebellitis. J Assoc Physicians India. 2016;64(2):69–70.

41. Bhoil R, Kumar S, Sood RG, Bhoil S, Verma R, Thakur R. Cerebellitis as an atypical manifestation of scrub typhus. *Neurology*. 2016;86(22):2113–4. doi: 10.1212/WNL.0000000000002723.

42. Premaratna R, Wijayalath SHNC, Miththinda JKND, Bandara NKBKRGW, de Silva HJ. Scrub typhus mimicking Parkinson's disease. *BMC Res Notes*. 2015;8:438. doi: 10.1186/s13104-015-1414-2.

43. Koti N, Mareddy AS, Nagri SK, Kudru CU. Dancing eyes and dancing feet in scrub typhus. *Australas Med J*. 2015;8(12):371–2. doi: 10.4066/AMJ.2015.2558.

44. Kim H-C, Yoon K-W, Yoo D-S, Cho C-S. Hemorrhagic transformation of scrub typhus encephalitis: A rare entity. *Clin Neuroradiol*. 2015;25(4):415–8. doi: 10.1007/s00062-014-0348-9.

45. Kim D-E, Cho G-Y, Oh Y-C, Choi S-M, Jung S-I, Lee S-H. Transient parkinsonism associated with scrub typhus. *J Korean Neurol Assoc*. 2015;33(2):126–8.

46. Bhat MD, Vykuntaraju KN, Acharya UV, Ramaswamy P, Prasad C. Isolated cerebellitis in scrub typhus. *Indian J Pediatr*. 2015;82(11):1067–8. doi: 10.1007/s12098-015-1772-4.

47. Karanth SS, Gupta A, Prabhu M. Pure cerebellitis due to scrub typhus: a unique case report. *Tropical Doctor*. 2013;43(1):41–2. doi:10.1177/0049475513480775.

48. Chiou YH, Yang CJ, Lai TH. Scrub typhus associated with transient parkinsonism and myoclonus. *Journal of Clinical Neuroscience*. 2013;20(1):182–3. doi:10.1016/j.jocn.2012.05.019.

49. Nam TS, Choi SM, Park KH, Kim MK, Cho KH. Opsoclonus associated with scrub typhus. *Neurology*. 2010;74(23):1925. doi:10.1212/WNL.0b013e3181e2422a.
