# Supplementary material for: Movement Disorders in Scrub Typhus: A Systematic Review
Source: Tremor Other Hyperkinet Mov (N Y). 2026 Mar 31;16:22. doi: 10.5334/tohm.1148 (PMC13045785; doi:10.5334/tohm.1148)
Supplement: Supplementary Table 2. — Summary of Cohort Studies Reporting Movement Disorders in Scrub Typhus. [file tohm-16-1-1148-s4.pdf]

Supplementary Table 2: Summary of Cohort Studies Reporting Movement Disorders in Scrub Typhus

Study (Author, Year)	Country	Total Patients with Scrub Typhus	Patients with Movement Disorder	Types of Movement Disorder	Number of Patients (Each)	Total Percentage (%)
Poongodi et al., 2025	India	50	9	Ataxia, Myoclonus, Cerebellitis (with ataxia)	4, 1, 4	18%
Majumdar et al., 2025	India	198	19	Tremor, Rigidity, Dyskinesias, Cerebellar ataxia, Opsoclonus-myoclonus with ataxia	8, 11, 4, 6, 2	9.6%
Rehani et al., 2024	India	7	1	Parkinsonism (symmetrical rigidity, bradykinesia, hypophonia)	1	14%
Gupta et al., 2024	India	105	2	Ataxia, Cerebellitis	2, 2	6.2%
Damodar et al., 2023	India	87	8	Opsoclonus–myoclonus, Choreoathetoid movements, Hemiballismus, Perioral dyskinesia, Lip smacking, Teeth grinding, Rapid eye blinking, Tremors	1, 1, 1, 1, 1, 1, 1, 2	9.3%
Saini et al., 2020	India	1 (6 children with opsoclonus myoclonus)	1	Opsoclonus, Ataxia	1, 1	100%
Ralph et al., 2019	India	1650	18	Opsoclonus, Myoclonus, Cerebellar dysfunction, Extrapyramidal syndrome (rigidity, tremor, parkinsonian gait)	18, 9, 8, 6	1.09%

Rana et al., 2017	India	323	4	Cerebellitis (ataxia)	4	1.2%
Lee et al., 2017	South Korea	16	1	Limb ataxia, Nystagmus, Dysarthria	1	6.25%

References

1. Poongodi R, Anandi C, Seenivasan M. Profile of Neurological Manifestations of Scrub Typhus in a Rural Tertiary Care Hospital. Research Journal of Medical Sciences. 2025;19:613-6. doi: 10.36478/makrjms.2025.1.613.616

2. Majumdar S, Benjamin RN, Ralph R, Mahasampath G, Abhilash KPP, Samson N, Sudarsan TI, Prakash JA, Varghese GM. Neurological Manifestations of Scrub Typhus: The Clinical Spectrum, Determinants, and Outcome. Neurol India. 2025;73(1):88-94. doi: 10.4103/ni.ni_139_22.

3. Rehani V, Sreen A, Anadure RK, Gupta S. The Spectrum of Neurological Manifestations in Scrub Typhus. Neurol India. 2024;72(3):610-614. doi: 10.4103/neuroindia.NI_470_20.

4. Gupta M, Tapaswini AP, Panda S, Sarangi R.Neurological manifestations of scrub typhus in children: Clinical profile and outcome. APIK J Int Med 2024;12:85-7. DOI: 10.4103/ajim.ajim_124_22

5. Damodar T, Singh B, Prabhu N, Marate S, Gowda VK, Lalitha AV, Dsouza FS, Sajjan SV, Kariyappa M, Kinhal UV, Prathyusha PV, Desai A, Thennarasu K, Solomon T, Ravi V, Yadav R. Association of Scrub Typhus in Children with Acute Encephalitis Syndrome and Meningoencephalitis, Southern India. Emerg Infect Dis. 2023;29(4):711-722. doi: 10.3201/eid2904.221157.

6. Saini L, Dhawan SR, Madaan P, Suthar R, Saini AG, Sahu JK, Sankhyan N. Infection-Associated Opsoclonus: A Retrospective Case Record Analysis and Review of Literature. J Child Neurol. 2020 Jun;35(7):480-484. doi: 10.1177/0883073820911327.

7. Ralph R, Prabhakar AT, Sathyendra S, Carey R, Jude J, Varghese GM. Scrub Typhus-Associated Opsoclonus: Clinical Course and Longitudinal Outcomes in an Indian Cohort. Ann Indian Acad Neurol. 2019;22(2):153-158. doi: 10.4103/aian.AIAN_198_18.

8. Rana A, Mahajan SK, Sharma A, Sharma S, Verma BS, Sharma A. Neurological manifestations of scrub typhus in adults. Trop Doct. 2017 Jan;47(1):22-25. doi: 10.1177/0049475516636543.

9. Lee HS, Sunwoo JS, Ahn SJ, Moon J, Lim JA, Jun JS, Lee WJ, Lee ST, Jung KH, Park KI, Jung KY, Lee SK, Chu K. Central Nervous System Infection Associated with Orientia tsutsugamushi in South Korea. Am J Trop Med Hyg. 2017 Oct;97(4):1094-1098. doi: 10.4269/ajtmh.17-0077.
